# Associations of Social Vulnerability and Race‐Ethnicity With Gastrointestinal Cancers in the United States

**DOI:** 10.1002/cam4.70591

**Published:** 2025-03-05

**Authors:** David J. Fei‐Zhang, David J. Bentrem, Jeffrey D. Wayne, Lifang Hou, Peiwen Fei, Timothy M. Pawlik

**Affiliations:** ^1^ Northwestern University Feinberg School of Medicine Chicago Illinois USA; ^2^ Department of Surgery Northwestern University Feinberg School of Medicine Chicago Illinois USA; ^3^ Department of Preventive Medicine Feinberg School of Medicine Chicago Illinois USA; ^4^ Center for Global Oncology Robert J. Havey, MD Institute for Global Health, Robert H. Lurie Comprehensive Cancer Center of Northwestern University Chicago Illinois USA; ^5^ University of Hawaii Cancer Center, University of Hawaii Honolulu Hawaii USA; ^6^ Department of Surgery, Division of Surgical Oncology The Ohio State University Wexner Medical Center and James Comprehensive Cancer Center Columbus Ohio USA

**Keywords:** disparity, ethnicity, gastrointestinal cancer, race, social determinants of health, social vulnerability

## Abstract

**Background:**

National social determinant of health (SDoH) studies on gastrointestinal cancers (GIC) have observed single GIC‐types for surgery but not across all GIC‐types, non‐surgical treatments outcomes, or mortality. The Social Vulnerability Index (SVI), a validated large‐data SDoH‐tool, quantifiably characterizes the interrelatedness of SDoH‐impact through dynamic, region‐contextualized measures.

**Methods:**

This retrospective cohort study assessed GIC patients (20+ years) between 2013 and 2017 from the Surveillance, Epidemiology, and End Results (SEER) database for total and subcomponent social vulnerability associations across 15 SDoH‐variables encompassing themes of socioeconomic status, minority‐language status, household composition, and housing‐transportation measured by the Social Vulnerability Index (SVI). These are measured and contextualized from all US counties. Univariate logistic and linear regressions of these vulnerability associations with treatment receipt (chemotherapy, radiation, primary surgery) and survival were performed for the entire cohort and across race/ethnicity strata.

**Results:**

With increasing overall social vulnerability, 287,248 patients (162,387 [56.5%] male; 185,250 [64.6%] white) demonstrated decreased receipt of chemotherapy (lowest, pancreas‐OR, 0.90; 95% CI, 0.88–0.93), radiotherapy (hepatic‐OR, 0.87; 95% CI, 0.85–0.89) and surgery (esophagus‐OR, 0.90; 95% CI, 0.87–0.92) for 13/14, 10/14, and 8/14 GIC‐types, respectively. Survival period decreases upwards of 21.3% (biliary tract: 6.9–5.4 months) were observed across 7/14 GICs. Treatment receipt and survival decreases were exacerbated for non‐White patients for 9/14 GICs. Socioeconomic status, minority‐language, household composition, and housing‐transportation vulnerabilities differentially contributed to these trends.

**Discussion:**

Social vulnerability was associated with worse prognostic and treatment disparities, with certain SDoH‐types differentially contributing to these detrimental trends per GIC‐type while associations were exacerbated among non‐White race/ethnic patients. These real‐world contexts present actionable targets for further initiatives to combat GIC disparities.

## Abstract 2

This social determinant of health (SDoH) study on 287,248 adult GIC‐patients showed worse multimodal treatment receipt and survival with increasing overall social vulnerability as measured by the Social Vulnerability Index (SVI) while quantifiably delineating which SDoH contribute more to GIC‐disparities.

## Graphical abstract

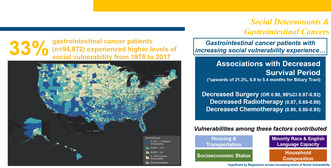

## 1 Introduction

With rising worldwide morbidity and mortality of gastrointestinal cancers (GIC), characterization of the factors associated with this detrimental trend has become a topic of increased interest [1, 2]. In addition to patient and clinical factors that contribute to a growing GIC disease burden, social determinants of health (SDoH) such as socioeconomic status, race‐ethnicity, transportation distance to a health provider, as well as other factors, have been identified as important drivers of inequalities in the diagnosis, delivery, and prognosis of patients. To this point, several studies have leveraged large‐data approaches to define the impact of SDoH‐related disparities relative to a range of disease processes [3, 4, 5].

While SDoH can impact a range of health care metrics, inequalities in social factors have disproportionate effects on patients receiving surgical treatment, especially among racial‐ethnicity minority patients [6, 7, 8, 9, 10]. In particular, the interactions between socioeconomic status and race‐ethnicity can have a modifying or synergistic effect on utilization and outcomes following surgical and non‐surgical interventions [3, 11]. For example, Bakkila et al. reported a decrease in chemotherapy and radiation therapy utilization among Black versus White patients with a GIC diagnosis [7]. Similarly, Reif de Paula et al. noted that adjuvant chemotherapy utilization differed among patients with pT4N0M0 colon cancer who had Medicare/Medicaid insurance or were uninsured [12]. Prior studies have primarily focused on race/ethnicity or insurance‐based disparities. However, SDoH‐related disparities related to non‐surgical treatment modalities for GICs have not been investigated at the national level.

In order to assess broader, real‐world implications of SDoH [3, 13, 14, 15], nuanced population‐based tools are needed to assess social factors associated with health care outcomes. The Centers for Disease Control and Prevention—Social Vulnerability Index (SVI) is a US census‐validated tool that measures community‐contextualized SDoH‐factors [16]. SVI encompasses themes of socioeconomic status (SES), minoritized race‐ethnicity and English‐language proficiency (ML), household composition (HH), and housing‐transportation status (HT). Prior studies have utilized the total SVI score to assess influence of overall social vulnerability with surgery receipt and post‐resection outcomes [13, 17, 18].

Characterization of how SDoH‐themes may differentially impact multi‐modal treatment and prognostic outcomes among patients with GIC has not been elucidated. Therefore, the objective of the current study was to define the association of SVI with utilization of surgical and non‐surgical treatment of GIC patients. We sought to examine how the different SVI subthemes (i.e., SES, ML, HH, and HT) may differentially affect treatment and prognosis of patients with GIC treated in the United States. Furthermore, we aimed to elucidate how the impact of the social vulnerability would differ among White and Non‐White patients diagnosed with GIC.

## 2 Methods

This retrospective cohort study followed the Strengthening the Reporting of Observational Studies in Epidemiology (STROBE) reporting guideline. The Northwestern University IRB deemed this research as exempt from needing ethics approvals or waiver of informed consent; the databases queried consist of publicly available, de‐identified data.

### 2.1 Data Sources and Variables

Data on SVI were extracted relative to ranked scores along fifteen census‐level social factors across four SDoH themes of socioeconomic status (poverty, unemployment, income level, high school diploma status), minority status‐language (minority status, proficiency with English), household composition (household members 65+ years, household members ≤ 17 years, disability status, single‐parent status), and housing‐transportation (multi‐unit structure, mobile homes, crowding, no vehicle, group quarters). Based on 2013–2018 CDC‐SVI documentation [16], SVI‐theme subscores were differentially weighed to formulate the total composite score and assigned different weights based on sociodemographic‐census data related to the designated area (Figure S1). Total and SVI‐theme scores were based on relative social vulnerabilities of a particular census tract among all 72,158 US‐census tracts, ranging from 0 to 1; 0 represented the lowest social vulnerability and 1 represented the highest. Full details regarding formulation and the differential weighing incorporated in SVI‐scores can be found in Data S1.

The National Cancer Institute‐Surveillance, Epidemiology, and End Results Program (NCI‐SEER) database contains datasets of patient variables, clinical characteristics, treatment modalities, and prognostic outcomes (see Data S1 for specific variables). SVI scores were matched to SEER‐patient data based on county of residence upon diagnosis. County‐assigned scores were generated by weighted score means per population density of each census tract within the county. A schematic workflow of this linkage is provided in Figure S2; descriptive details regarding this process of geocode linkage can be found in Data S1.

### 2.2 Population Definitions

The SEER database was queried for adult (> 20 years) patients diagnosed with GIC between 2013 and 2017 using International Classification of Diseases for Oncology, Third Edition (ICD‐O‐3) topographic codes [C15.0—C26.9]. Gastrointestinal stromal tumors (GIST) and neuroendocrine tumors (NET) identified based on SEER‐generated histologic categories were excluded to homogenize the histological representation of primary site tumors.

Missing data entries due to lacking geographical information allowing SVI‐variable linkage were excluded from all analyses. Unavailable data for a specified outcome necessitated for an analysis were excluded solely for that respective calculation. Survival/mortality was considered as “all‐cause mortality” per SEER‐data documentation.

### 2.3 Statistical Methods

SVI scores were categorized into quintiles based on SVI scores within each disease class. The relative‐SVI quintiles were defined as “< 20”, “20–39.99”, “40–59.99”, “60–79.99”, “80–99.99”, which represented the relative percentiles per disease class (e.g., within a certain GIC diagnosis, patients with the lowest SVI scores are grouped into the “< 20” quintile group). Indicated chemotherapy, radiotherapy, and primary surgical resection within each GIC‐type were analyzed using univariate logistic regression across the relative‐SVI quintiles (representative of the differentially weighted aggregate of the 15 SDoH‐variables), as well as each of the four SVI themes.

In assessing the impact of overall SVI and SVI‐theme quintiles, differences in the mean months of survival for lowest and highest SVI‐scored quintiles were calculated within each GIC type. Trend significance was assessed by linear regression across all data points relative to SVI quintiles for months survival. For each relative‐SVI quintile, the median, interquartile range (IQR), and 1.5‐times the IQR (i.e., boxplot) was calculated. Means, standard deviations, and ranges for months survival per quintile were also calculated across GIC types. In addition, Kaplan–Meier survival analyses of the entire GIC cohort with log‐rank significance testing was also conducted between the lowest and highest total SVI quintile groups.

Odds of receiving a certain treatment were analyzed with univariate logistic regressions across relative‐SVI quintiles for increasing levels of total SVI and each SVI‐theme. References groups for odds ratios and 95% confidence intervals calculated for each SVI‐association were represented by the lowest relative SVI‐quintile (i.e., lowest social vulnerability group) with subsequent ordinal variable values sequentially matching each increasing level of relative quintile (i.e., “< 20” quintile was set as the reference, increasing levels of comparator values were ordinally organized by “20–39.99”, “40–59.99”, “60–79.99”, and “80–99.99” in sequence). Binary outcome delineations for outcome variables were utilized for whether a patient received surgical resection, radiation therapy, or chemotherapy. For race‐ethnicity stratified analyses, similar techniques were performed independently regarding months of survival and treatment receipt within cohorts of patients that were (Non‐Hispanic) White and Non‐White (Hispanic, Black, Asian or Pacific Islander, Native American, or Other).

Statistical significance was set as *p* < 0.05. Two‐sided *p*‐values were reported for analyses. All statistical analyses were conducted in R version 4.2.1.

## 3 Results

Among 287,248 patients with GIC who were included in the analytic cohort, most patients were between 65 and 84 years of age (*n* = 137,674, 47.9%), male (*n* = 162,387, 56.5%), and self‐identified as non‐Hispanic White (*n* = 185,450, 64.6%) (Table 1). The primary GIC diagnosis varied considerably within the study population (esophagus, *n* = 16,276, 5.6%; gastroesophageal junction, *n* = 7961, 2.8%; stomach, *n* = 15,261, 5.3%; liver, *n* = 31,105, 10.8%; pancreas, *n* = 49,798, 17.3%; biliary tract, *n* = 10,510, 3.6%; gallbladder, *n* = 4550, 1.6%; small intestine, *n* = 3075, 1.1%; colon, *n* = 97,990, 34.1%; rectum, *n* = 40,351, 14.0%; anus, *n* = 7274, 2.5%; gastrointestinal‐other, *n* = 3097, 1.1%). In assessing social vulnerability, total SVI scores ranged from 0.000 to 0.947 (subthemes: socioeconomic status‐SVI: 0.000–0.976; minority status and language‐SVI: 0.002–0.945; household composition‐SVI: 0.091–0.971; housing and transport‐SVI: 0.051–0.942) (Table 1). Treatment of patients with GIC also varied (chemotherapy, *n* = 123,822, 43.1%; radiation therapy, *n* = 50,103, 17.4%; surgical resection, *n* = 146,487, 52.9%) (Table 1). Clinicodemographic distribution across each SVI‐subtheme was also varied (Tables [Link], [Link], [Link], [Link]).

**TABLE 1:** Patient characteristics by Total SVI Score.

		Total SVI Score					*p*
Characteristic	Overall, *N* = 287,248 (100%)	0.000–0.199, *N* = 2238 (0.8%)	0.200–0.399, *N* = 52,888 (18%)	0.400–0.599, *N* = 137,250 (48%)	0.600–0.799, *N* = 88,548 (31%)	0.800–0.999, *N* = 6324 (2.2%)	
Age							< 0.001
20–44 years	12,110 (4.2%)	92 (4.1%)	2138 (4.0%)	5667 (4.1%)	3956 (4.5%)	257 (4.1%)	
45–64 years	105,661 (37%)	802 (36%)	18,178 (34%)	50,629 (37%)	33,628 (38%)	2424 (38%)	
65–84 years	137,674 (48%)	1108 (50%)	25,947 (49%)	65,374 (48%)	42,115 (48%)	3130 (49%)	
85+ years	31,803 (11%)	236 (11%)	6625 (13%)	15,580 (11%)	8849 (10.0%)	513 (8.1%)	
Sex							< 0.001
Male	162,387 (57%)	1289 (58%)	29,484 (56%)	77,622 (57%)	50,295 (57%)	3697 (58%)	
Female	124,861 (43%)	949 (42%)	23,404 (44%)	59,628 (43%)	38,253 (43%)	2627 (42%)	
Race							< 0.001
White	185,450 (65%)	1928 (86%)	43,799 (83%)	87,604 (64%)	48,714 (55%)	3405 (54%)	
Hispanic	37,956 (13%)	180 (8.0%)	2640 (5.0%)	14,144 (10%)	19,397 (22%)	1595 (25%)	
Black	34,239 (12%)	40 (1.8%)	3578 (6.8%)	18,064 (13%)	11,630 (13%)	927 (15%)	
Asian or Pacific Islander	26,267 (9.1%)	43 (1.9%)	2440 (4.6%)	15,994 (12%)	7671 (8.7%)	119 (1.9%)	
Native American	1866 (0.6%)	32 (1.4%)	194 (0.4%)	756 (0.6%)	624 (0.7%)	260 (4.1%)	
Unknown	1470 (0.5%)	15 (0.7%)	237 (0.4%)	688 (0.5%)	512 (0.6%)	18 (0.3%)	
Region							< 0.001
Midwest	26,674 (9.3%)	145 (6.5%)	9788 (19%)	16,170 (12%)	571 (0.6%)	0 (0%)	
Northeast	45,747 (16%)	1021 (46%)	21,655 (41%)	17,825 (13%)	5246 (5.9%)	0 (0%)	
South	66,701 (23%)	779 (35%)	7730 (15%)	32,662 (24%)	22,925 (26%)	2605 (41%)	
West	148,126 (52%)	293 (13%)	13,715 (26%)	70,593 (51%)	59,806 (68%)	3719 (59%)	
Primary site							< 0.001
Anus	7274 (2.5%)	70 (3.1%)	1386 (2.6%)	3570 (2.6%)	2127 (2.4%)	121 (1.9%)	
Biliary tract	10,510 (3.7%)	104 (4.6%)	2022 (3.8%)	5041 (3.7%)	3101 (3.5%)	242 (3.8%)	
Colon	97,990 (34%)	766 (34%)	18,130 (34%)	46,369 (34%)	30,470 (34%)	2255 (36%)	
Esophagus	16,276 (5.7%)	148 (6.6%)	3384 (6.4%)	7926 (5.8%)	4464 (5.0%)	354 (5.6%)	
Gallbladder	4550 (1.6%)	24 (1.1%)	787 (1.5%)	2146 (1.6%)	1490 (1.7%)	103 (1.6%)	
Gastroesophageal junction	7961 (2.8%)	69 (3.1%)	1652 (3.1%)	3854 (2.8%)	2255 (2.5%)	131 (2.1%)	
Gastrointestinal, other	3097 (1.1%)	20 (0.9%)	611 (1.2%)	1392 (1.0%)	1017 (1.1%)	57 (0.9%)	
Liver	31,105 (11%)	177 (7.9%)	4554 (8.6%)	15,602 (11%)	10,020 (11%)	752 (12%)	
Pancreas, other	13,369 (4.7%)	115 (5.1%)	2645 (5.0%)	6296 (4.6%)	4043 (4.6%)	270 (4.3%)	
Pancreatic body and tail	13,860 (4.8%)	112 (5.0%)	2879 (5.4%)	6792 (4.9%)	3828 (4.3%)	249 (3.9%)	
Pancreatic head	22,569 (7.9%)	202 (9.0%)	4436 (8.4%)	10,944 (8.0%)	6539 (7.4%)	448 (7.1%)	
Rectum	40,351 (14%)	327 (15%)	7561 (14%)	18,829 (14%)	12,699 (14%)	935 (15%)	
Small intestine	3075 (1.1%)	27 (1.2%)	626 (1.2%)	1464 (1.1%)	905 (1.0%)	53 (0.8%)	
Stomach	15,261 (5.3%)	77 (3.4%)	2215 (4.2%)	7025 (5.1%)	5590 (6.3%)	354 (5.6%)	
TNM/AJCC Combined stage							0.010
Stage I‐III	175,818 (67%)	1347 (66%)	32,333 (67%)	85,053 (67%)	53,271 (67%)	3814 (67%)	
Stage IV and Above	86,936 (33%)	706 (34%)	16,257 (33%)	41,451 (33%)	26,659 (33%)	1863 (33%)	
Primary surgery performed							< 0.001
No surgery	130,562 (47%)	1014 (47%)	23,766 (46%)	62,279 (47%)	40,600 (48%)	2903 (48%)	
Surgery	146,487 (53%)	1145 (53%)	27,503 (54%)	70,301 (53%)	44,406 (52%)	3132 (52%)	
Radiation therapy performed							< 0.001
No therapy	237,145 (83%)	1826 (82%)	42,910 (81%)	112,703 (82%)	74,464 (84%)	5242 (83%)	
Therapy	50,103 (17%)	412 (18%)	9978 (19%)	24,547 (18%)	14,084 (16%)	1082 (17%)	
Chemotherapy performed							< 0.001
No therapy	163,426 (57%)	1129 (50%)	28,868 (55%)	77,034 (56%)	52,624 (59%)	3771 (60%)	
Therapy	123,822 (43%)	1109 (50%)	24,020 (45%)	60,216 (44%)	35,924 (41%)	2553 (40%)	
Vital status on last follow‐up							< 0.001
Alive	153,472 (53%)	1261 (56%)	29,152 (55%)	73,677 (54%)	46,221 (52%)	3161 (50%)	
Dead	133,776 (47%)	977 (44%)	23,736 (45%)	63,573 (46%)	42,327 (48%)	3163 (50%)	

### 3.1 Impact of SVI on Treatment of GIC

**FIGURE 1:**
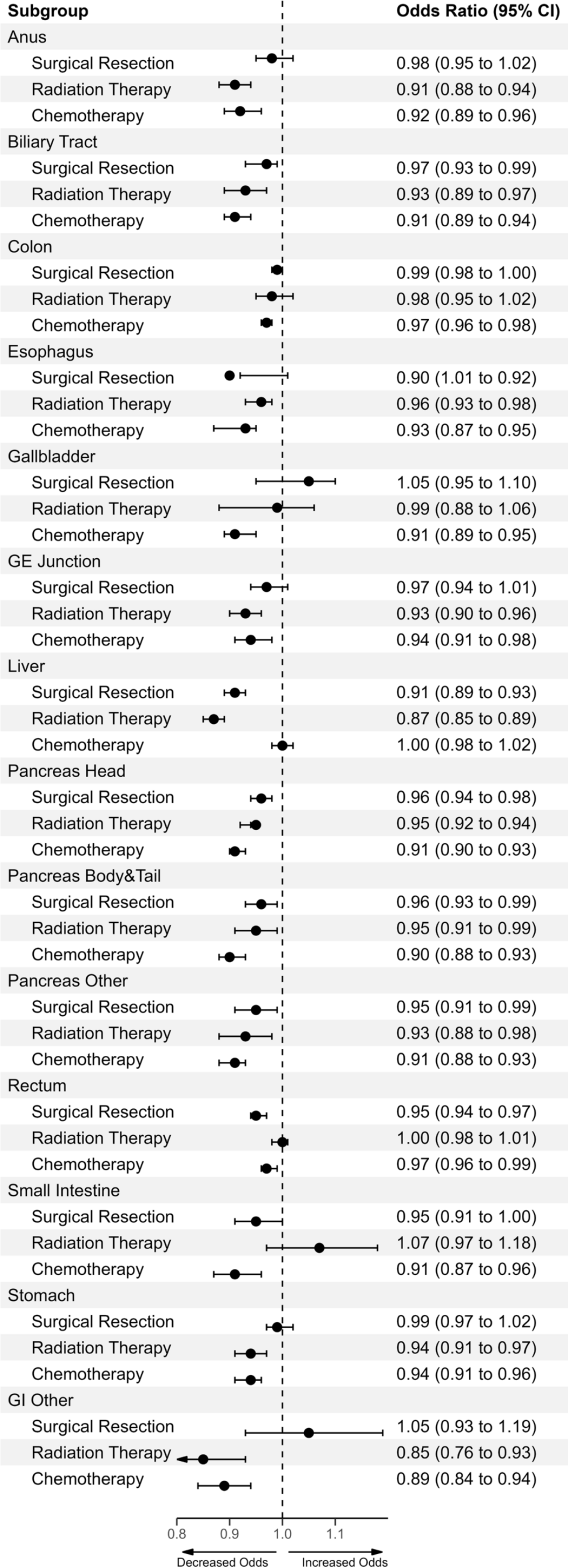
Treatment receipt with increasing total SVI Scores. Univariate logistic regressions across increasing total SVI quintiles based on indicated surgery, radiation treatment, and chemotherapy receipt per primary site.

Social vulnerability was strongly associated with likelihood to receive chemotherapy, radiation therapy, as well as surgical resection. Specifically, across many GIC diagnoses, patients residing in counties with higher total SVI scores had decreased odds of receiving surgical resection on 8/14 GIC types (lowest, esophagus: OR, 0.90; 95% CI, 0.87–0.92) (see Data S1 for all significant sites) (Figure 1, Table 2). Of note, the subthemes that had the largest impact on decreased likelihood to undergo surgical resection included increasing vulnerability in household composition and socioeconomic status, followed by housing‐transportation and then minority‐language status (Table 2).

**TABLE 2:** Treatment receipt with increasing SVI Scores.

Primary classification^a^	Characteristic	Surgical resection			Radiation therapy			Chemotherapy		
		OR	95% CI	*p*	OR	95% CI	*p*	OR	95% CI	*p*
Anus	Total	0.98	0.95, 1.02	0.366	0.91	0.88, 0.94	< 0.001	0.92	0.89, 0.96	< 0.001
	Socioeconomic status	1.00	0.96, 1.03	0.803	0.93	0.90, 0.97	< 0.001	0.93	0.90, 0.97	< 0.001
	Minority‐language status	0.99	0.96, 1.02	0.479	0.90	0.87, 0.94	< 0.001	0.93	0.90, 0.97	< 0.001
	Household composition	1.00	0.96, 1.03	0.907	1.04	1.00, 1.07	0.071	1.01	0.97, 1.04	0.774
	Housing‐transportation	0.98	0.95, 1.01	0.191	0.92	0.89, 0.96	< 0.001	0.95	0.91, 0.98	0.003
Biliary tract	Total	0.97	0.93, 0.99	0.042	0.93	0.89, 0.97	0.001	0.91	0.89, 0.94	< 0.001
	Socioeconomic status	0.95	0.92, 0.98	0.003	0.96	0.92, 1.00	0.045	0.94	0.91, 0.96	< 0.001
	Minority‐language status	1.05	1.01, 1.08	0.010	0.92	0.88, 0.96	< 0.001	0.94	0.91, 0.96	< 0.001
	Household composition	0.92	0.89, 0.95	< 0.001	1.03	0.99, 1.08	0.096	0.98	0.96, 1.01	0.265
	Housing‐transportation	0.96	0.93, 0.99	0.014	0.89	0.86, 0.93	< 0.001	0.91	0.89, 0.94	< 0.001
Colon	Total	0.99	0.98, 1.00	0.118	0.98	0.95, 1.02	0.339	0.97	0.96, 0.98	< 0.001
	Socioeconomic status	1.0	0.98, 1.01	0.393	1.01	0.97, 1.04	0.630	0.98	0.97, 0.99	< 0.001
	Minority‐language status	0.97	0.96, 0.98	< 0.001	0.90	0.87, 0.94	< 0.001	0.96	0.95, 0.97	< 0.001
	Household composition	1.02	1.01, 1.04	< 0.001	1.07	1.04, 1.11	< 0.001	1.00	1.0, 1.01	0.380
	Housing‐transportation	0.99	0.98, 1.00	0.063	0.98	0.94, 1.01	0.193	0.97	0.96, 0.98	< 0.001
Esophagus	Total	0.90	0.87, 0.92	< 0.001	0.96	0.93, 0.98	< 0.001	0.93	0.90, 0.95	< 0.001
	Socioeconomic status	0.91	0.88, 0.93	< 0.001	0.97	0.95, 0.99	0.010	0.94	0.92, 0.96	< 0.001
	Minority‐language status	0.93	0.91, 0.96	< 0.001	0.94	0.92, 0.96	< 0.001	0.94	0.92, 0.96	< 0.001
	Household composition	0.96	0.93, 0.98	0.001	1.00	0.98, 1.03	0.776	0.98	0.96, 1.01	0.159
	Housing‐transportation	0.94	0.91, 0.96	< 0.001	0.97	0.95, 0.99	0.006	0.94	0.92, 0.96	< 0.001
Gallbladder	Total	1.05	1.01, 1.10	0.024	0.99	0.93, 1.06	0.843	0.91	0.87, 0.95	< 0.001
	Socioeconomic status	1.06	1.02, 1.11	0.006	1.00	0.94, 1.07	0.917	0.92	0.88, 0.96	< 0.001
	Minority‐language status	0.99	0.95, 1.03	0.576	0.95	0.89, 1.01	0.101	0.94	0.90, 0.98	0.003
	Household composition	1.02	0.98, 1.06	0.422	1.04	0.98, 1.11	0.227	0.98	0.94, 1.02	0.295
	Housing‐transportation	1.02	0.98, 1.07	0.272	0.98	0.92, 1.05	0.598	0.92	0.88, 0.96	< 0.001
Gastroesophageal junction	Total	0.97	0.94, 1.01	0.106	0.93	0.90, 0.96	< 0.001	0.94	0.91, 0.98	< 0.001
	Socioeconomic status	0.98	0.95, 1.01	0.223	0.98	0.95, 1.01	0.123	0.94	0.91, 0.97	< 0.001
	Minority‐language status	0.98	0.94, 1.01	0.139	0.88	0.85, 0.90	< 0.001	0.96	0.93, 0.99	0.015
	Household composition	1.01	0.98, 1.04	0.539	1.07	1.04, 1.11	< 0.001	0.97	0.94, 1.01	0.099
	Housing‐transportation	0.98	0.95, 1.01	0.277	0.94	0.91, 0.97	< 0.001	0.96	0.93, 0.99	0.007
Liver	Total	0.91	0.89, 0.93	< 0.001	0.87	0.85, 0.89	< 0.001	1.00	0.98, 1.02	0.960
	Socioeconomic status	0.89	0.87, 0.90	< 0.001	0.92	0.89, 0.94	< 0.001	1.00	0.98, 1.01	0.776
	Minority‐language status	1.00	0.98, 1.02	0.868	0.83	0.81, 0.86	< 0.001	1.02	1.00, 1.04	0.029
	Household composition	0.89	0.87, 0.91	< 0.001	1.04	1.01, 1.07	0.003	1.00	0.99, 1.02	0.588
	Housing‐transportation	0.95	0.94, 0.97	< 0.001	0.85	0.83, 0.88	< 0.001	0.98	0.97, 1.00	0.053
Pancreatic head	Total	0.96	0.94, 0.98	< 0.001	0.95	0.92, 0.97	< 0.001	0.91	0.90, 0.93	< 0.001
	Socioeconomic status	0.94	0.92, 0.96	< 0.001	1.00	0.97, 1.02	0.879	0.93	0.92, 0.95	< 0.001
	Minority‐language status	1.02	1.00, 1.04	0.099	0.88	0.86, 0.90	< 0.001	0.94	0.92, 0.95	< 0.001
	Household composition	0.95	0.93, 0.97	< 0.001	1.08	1.05, 1.10	< 0.001	0.98	0.97, 1.00	0.078
	Housing‐transportation	0.99	0.97, 1.01	0.231	0.91	0.88, 0.93	< 0.001	0.91	0.89, 0.93	< 0.001
Pancreatic body and tail	Total	0.96	0.93, 0.99	0.008	0.95	0.91, 0.99	0.013	0.90	0.88, 0.93	< 0.001
	Socioeconomic status	0.95	0.92, 0.97	< 0.001	0.98	0.94, 1.03	0.452	0.93	0.91, 0.95	< 0.001
	Minority‐language status	1.05	1.02, 1.08	0.001	0.90	0.86, 0.94	< 0.001	0.94	0.92, 0.96	< 0.001
	Household composition	0.93	0.91, 0.96	< 0.001	1.04	0.99, 1.08	0.108	0.96	0.94, 0.98	0.001
	Housing‐transportation	0.98	0.95, 1.01	0.169	0.92	0.88, 0.96	< 0.001	0.91	0.89, 0.94	< 0.001
Pancreatic, other	Total	0.95	0.91, 0.99	0.016	0.93	0.88, 0.98	0.004	0.91	0.88, 0.93	< 0.001
	Socioeconomic status	0.92	0.89, 0.97	< 0.001	0.95	0.91, 1.00	0.066	0.92	0.89, 0.94	< 0.001
	Minority‐language status	1.03	0.99, 1.08	0.179	0.92	0.87, 0.96	0.001	0.97	0.95, 1.00	0.039
	Household composition	0.93	0.89, 0.97	0.001	1.00	0.95, 1.05	0.969	0.94	0.91, 0.96	< 0.001
	Housing‐transportation	0.97	0.93, 1.01	0.134	0.90	0.85, 0.94	< 0.001	0.93	0.91, 0.95	< 0.001
Rectum	Total	0.95	0.94, 0.97	< 0.001	1.00	0.98, 1.01	0.564	0.97	0.96, 0.99	< 0.001
	Socioeconomic status	0.96	0.95, 0.98	< 0.001	1.01	1.00, 1.03	0.106	0.99	0.97, 1.00	0.043
	Minority‐language status	0.94	0.93, 0.96	< 0.001	0.95	0.93, 0.96	< 0.001	0.95	0.93, 0.96	< 0.001
	Household composition	1.00	0.99, 1.02	0.769	1.05	1.04, 1.07	< 0.001	1.02	1.01, 1.04	0.001
	Housing‐transportation	0.96	0.95, 0.98	< 0.001	1.00	0.98, 1.01	0.539	0.98	0.96, 0.99	0.002
Small intestine	Total	0.95	0.91, 1.00	0.066	1.07	0.97, 1.18	0.205	0.91	0.87, 0.96	0.001
	Socioeconomic status	0.96	0.91, 1.01	0.099	1.00	0.90, 1.10	0.924	0.95	0.90, 1.0	0.030
	Minority‐language status	0.95	0.90, 1.00	0.032	1.04	0.95, 1.15	0.380	0.96	0.91, 1.01	0.136
	Household composition	1.02	0.97, 1.07	0.486	1.02	0.93, 1.13	0.675	0.96	0.92, 1.02	0.166
	Housing‐transportation	0.94	0.90, 0.99	0.022	1.07	0.97, 1.18	0.183	0.90	0.86, 0.95	< 0.001
Stomach	Total	0.99	0.97, 1.02	0.567	0.94	0.91, 0.97	< 0.001	0.94	0.91, 0.96	< 0.001
	Socioeconomic status	0.97	0.95, 0.99	0.006	0.96	0.93, 0.99	0.007	0.93	0.91, 0.96	< 0.001
	Minority‐language status	1.04	1.01, 1.06	0.003	0.91	0.88, 0.94	< 0.001	0.98	0.96, 1.00	0.049
	Household composition	0.95	0.93, 0.98	< 0.001	1.02	0.99, 1.05	0.196	0.95	0.93, 0.97	< 0.001
	Housing‐transportation	1.01	0.99, 1.04	0.322	0.96	0.93, 0.99	0.007	0.97	0.94, 0.99	0.004
Gastrointestinal, other	Total	1.05	0.93, 1.19	0.404	0.85	0.76, 0.93	0.001	0.89	0.84, 0.94	< 0.001
	Socioeconomic status	1.06	0.94, 1.20	0.341	0.87	0.79, 0.96	0.006	0.93	0.87, 0.98	0.006
	Minority‐language status	1.07	0.95, 1.21	0.252	0.89	0.80, 0.98	0.017	0.91	0.86, 0.96	0.001
	Household composition	1.00	0.89, 1.13	0.965	0.97	0.88, 1.07	0.497	1.01	0.96, 1.07	0.603
	Housing‐transportation	0.93	0.82, 1.05	0.250	0.91	0.82, 1.00	0.062	0.86	0.81, 0.91	< 0.001

^a^ By American Joint Committee on Cancer, 6th Edition (AJCC‐6); Univariate logistic regressions with calculated Odds Ratios (OR) and 95% confidence intervals (95% CI) were performed with lowest relative SVI quintiles set as the reference within each histopathology type.

Patients residing in counties with higher total SVI scores also had decreased odds of receiving chemotherapy (lowest, pancreas‐OR, 0.90; 95% CI, 0.88–0.93) (see Data S1 for all significant sites) (Figure 1, Table 2). Similarly, patients with higher total SVI also had decreased odds of receiving radiation therapy (lowest, hepatic‐OR, 0.87; 95% CI, 0.85–0.89) (see Data S1 for all significant sites) (Figure 1, Table 2). Of note, the subthemes that had the biggest impact on decreased likelihood to receive chemotherapy included increasing vulnerability in housing‐transportation, followed by minority‐language status and socioeconomic status, as well as household composition proportionally. In contrast, the vulnerability subthemes with the biggest impact on radiation therapy receipt included vulnerability in minority‐language status and socioeconomic status, with household composition and housing‐transportation proportionally contributing the same impact (Table 2).

**FIGURE 2:**
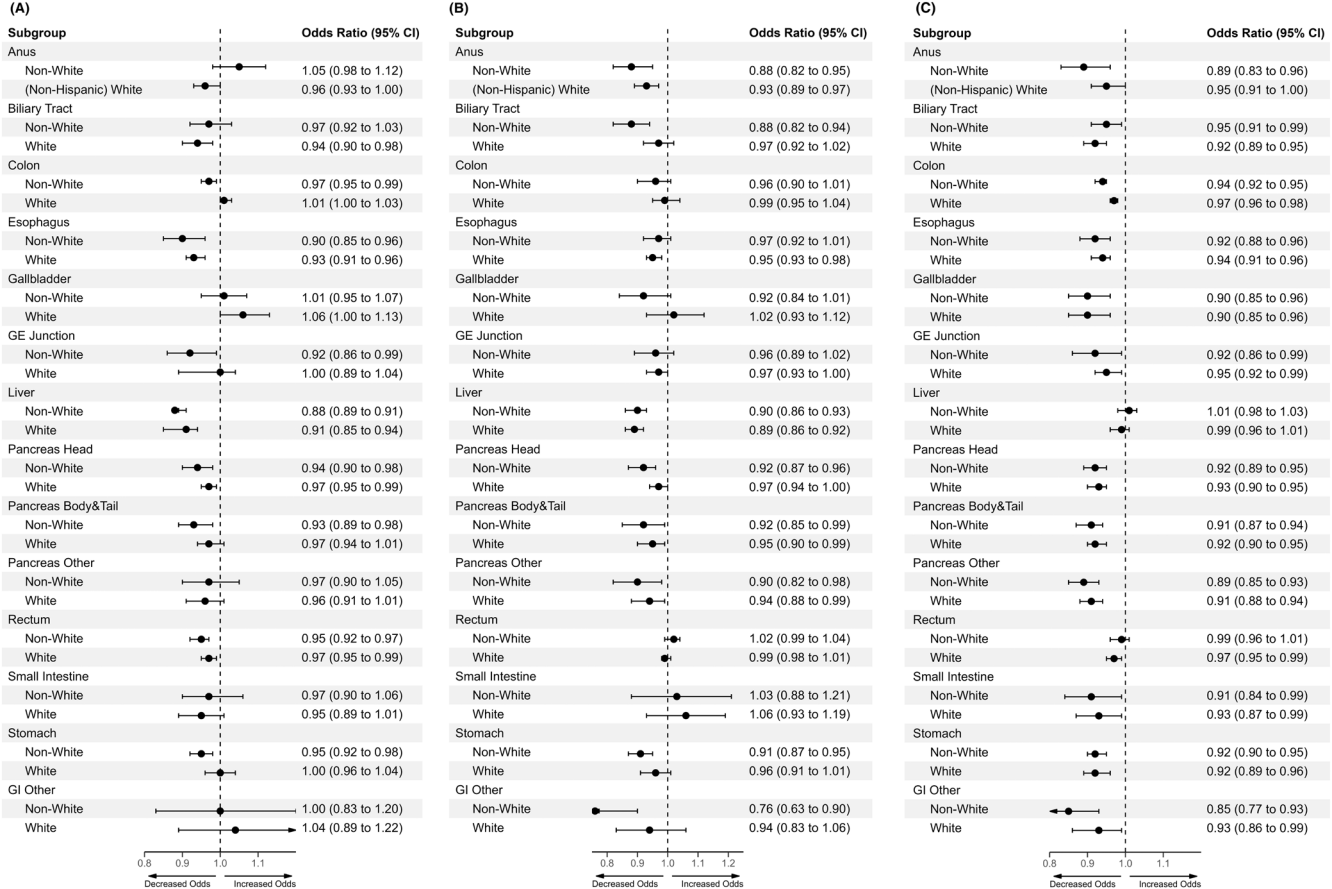
Race/ethnicity‐stratified multimodal treatment receipt with increasing total SVI Scores. Across Non‐White and (Non‐Hispanic) White GIC‐patient cohorts, univariate logistic regressions across increasing total SVI quintiles based on indicated (A) surgery, (B) radiation treatment, (C) chemotherapy receipt per primary site.

When differentiating the influence of increasing county‐level social vulnerabilities between White and Non‐White GIC patient cohorts, both cohorts displayed decreased odds of receiving indicated surgical resection and radiation therapy across several GICs. However, Non‐White patients displayed higher‐magnitude decreases compared to White patients across different GIC types for each modality (Figure 2, Table 3).

**TABLE 3:** Race/ethnicity‐stratified multimodal treatment receipt with increasing SVI Scores.

Primary classification^a^	Characteristic	Surgical resection			Radiation therapy			Chemotherapy		
		OR	95% CI	*p*	OR	95% CI	*p*	OR	95% CI	*p*
Anus—Non‐White	Total	1.05	0.98, 1.12	0.200	0.88	0.82, 0.95	0.001	0.89	0.83, 0.96	0.002
	Socioeconomic status	1.06	0.99, 1.14	0.077	0.91	0.85, 0.98	0.017	0.89	0.83, 0.96	0.002
	Minority‐language status	0.98	0.91, 1.05	0.525	0.91	0.85, 0.98	0.017	0.93	0.87, 1.00	0.054
	Household composition	1.04	0.97, 1.12	0.224	1.00	0.93, 1.08	0.997	0.97	0.90, 1.04	0.412
	Housing‐transportation	1.00	0.93, 1.07	0.912	0.93	0.86, 1.00	0.051	0.97	0.90, 1.04	0.389
Anus—White	Total	0.96	0.93, 1.00	0.056	0.93	0.89, 0.97	0.001	0.95	0.91, 1.00	0.033
	Socioeconomic status	0.97	0.94, 1.01	0.166	0.94	0.90, 0.99	0.010	0.96	0.92, 1.00	0.056
	Minority‐language status	0.98	0.94, 1.01	0.214	0.91	0.87, 0.95	< 0.001	0.95	0.91, 0.99	0.023
	Household composition	0.99	0.95, 1.03	0.621	1.03	0.99, 1.08	0.173	1.00	0.96, 1.05	0.844
	Housing‐transportation	0.96	0.93, 1.00	0.064	0.92	0.88, 0.96	< 0.001	0.95	0.91, 0.99	0.013
Biliary tract—Non‐White	Total	0.97	0.92, 1.03	0.292	0.88	0.82, 0.94	< 0.001	0.95	0.91, 0.99	0.026
	Socioeconomic status	0.95	0.90, 1.01	0.100	0.91	0.84, 0.97	0.008	0.98	0.93, 1.02	0.298
	Minority‐language status	1.05	1.00, 1.11	0.069	0.92	0.85, 0.99	0.020	0.95	0.91, 1.00	0.037
	Household composition	0.91	0.86, 0.96	< 0.001	0.99	0.92, 1.06	0.760	1.03	0.98, 1.07	0.264
	Housing‐transportation	1.00	0.94, 1.05	0.915	0.86	0.80, 0.93	< 0.001	0.92	0.88, 0.97	0.001
Biliary tract—White	Total	0.94	0.90, 0.98	0.007	0.97	0.92, 1.02	0.196	0.92	0.89, 0.95	< 0.001
	Socioeconomic status	0.95	0.91, 0.99	0.011	1.01	0.96, 1.06	0.781	0.93	0.89, 0.96	< 0.001
	Minority‐language status	1.05	1.00, 1.09	0.030	0.96	0.91, 1.01	0.098	0.95	0.92, 0.98	0.004
	Household composition	0.92	0.88, 0.96	< 0.001	1.04	0.99, 1.09	0.099	0.96	0.93, 0.99	0.018
	Housing‐transportation	0.95	0.91, 0.99	0.011	0.93	0.89, 0.98	0.006	0.94	0.91, 0.97	< 0.001
Colon—Non‐White	Total	0.97	0.95, 0.99	0.005	0.96	0.90, 1.01	0.133	0.94	0.92, 0.95	< 0.001
	Socioeconomic status	0.97	0.95, 0.99	0.003	0.97	0.92, 1.03	0.382	0.95	0.94, 0.97	< 0.001
	Minority‐language status	1.00	0.98, 1.02	0.869	0.92	0.86, 0.97	0.004	0.91	0.90, 0.93	< 0.001
	Household composition	0.99	0.97, 1.01	0.171	1.03	0.97, 1.09	0.330	1.00	0.98, 1.02	0.971
	Housing‐transportation	1.00	0.98, 1.02	0.806	0.96	0.91, 1.02	0.213	0.93	0.92, 0.95	< 0.001
Colon—White	Total	1.01	1.00, 1.03	0.158	0.99	0.95, 1.04	0.782	0.97	0.96, 0.98	< 0.001
	Socioeconomic status	1.02	1.00, 1.03	0.039	1.02	0.98, 1.07	0.295	0.98	0.97, 0.99	0.002
	Minority‐language status	0.97	0.96, 0.99	< 0.001	0.89	0.86, 0.93	< 0.001	0.95	0.94, 0.96	< 0.001
	Household composition	1.04	1.02, 1.05	< 0.001	1.10	1.05, 1.14	< 0.001	1.01	1.0, 1.02	0.267
	Housing‐transportation	1.00	0.99, 1.02	0.656	0.98	0.94, 1.02	0.394	0.96	0.95, 0.98	< 0.001
Esophagus—Non‐White	Total	0.90	0.85, 0.96	0.002	0.97	0.92, 1.01	0.151	0.92	0.88, 0.96	< 0.001
	Socioeconomic status	0.89	0.83, 0.95	< 0.001	0.97	0.93, 1.02	0.209	0.93	0.89, 0.98	0.003
	Minority‐language status	1.07	1.00, 1.14	0.043	0.89	0.85, 0.93	< 0.001	0.93	0.89, 0.97	0.001
	Household composition	0.87	0.82, 0.93	< 0.001	1.04	0.99, 1.08	0.131	0.99	0.95, 1.04	0.765
	Housing‐transportation	1.00	0.94, 1.07	0.999	0.96	0.92, 1.00	0.080	0.93	0.89, 0.98	0.003
Esophagus—White	Total	0.93	0.91, 0.96	< 0.001	0.95	0.93, 0.98	< 0.001	0.94	0.91, 0.96	< 0.001
	Socioeconomic status	0.93	0.90, 0.96	< 0.001	0.96	0.94, 0.99	0.004	0.95	0.92, 0.97	< 0.001
	Minority‐language status	0.95	0.92, 0.98	0.001	0.95	0.92, 0.97	< 0.001	0.95	0.93, 0.98	< 0.001
	Household composition	0.97	0.94, 1.00	0.047	0.99	0.97, 1.02	0.598	0.98	0.95, 1.00	0.087
	Housing‐transportation	0.96	0.93, 0.99	0.003	0.97	0.95, 1.00	0.046	0.95	0.93, 0.98	< 0.001
Gallbladder—Non‐White	Total	1.01	0.95, 1.07	0.799	0.92	0.84, 1.01	0.089	0.90	0.85, 0.96	0.001
	Socioeconomic status	1.00	0.94, 1.07	0.898	0.97	0.89, 1.06	0.490	0.91	0.86, 0.97	0.003
	Minority‐language status	1.00	0.94, 1.06	0.958	0.88	0.80, 0.96	0.004	0.89	0.84, 0.95	< 0.001
	Household composition	0.98	0.92, 1.04	0.546	1.09	0.99, 1.19	0.066	0.99	0.93, 1.05	0.769
	Housing‐transportation	1.02	0.96, 1.09	0.456	0.93	0.85, 1.02	0.114	0.89	0.84, 0.95	< 0.001
Gallbladder—White	Total	1.06	1.00, 1.13	0.039	1.02	0.93, 1.12	0.653	0.90	0.85, 0.96	0.001
	Socioeconomic status	1.11	1.04, 1.17	0.001	1.03	0.94, 1.12	0.581	0.92	0.87, 0.98	0.006
	Minority‐language status	0.96	0.90, 1.02	0.157	0.98	0.89, 1.07	0.619	0.95	0.89, 1.00	0.067
	Household composition	1.05	0.99, 1.12	0.079	1.03	0.94, 1.12	0.576	0.97	0.91, 1.02	0.253
	Housing‐transportation	1.03	0.97, 1.09	0.411	1.01	0.93, 1.11	0.756	0.91	0.86, 0.97	0.003
Gastroesophageal junction—Non‐White	Total	0.92	0.86, 0.99	0.025	0.96	0.89, 1.02	0.201	0.92	0.86, 0.99	0.016
	Socioeconomic status	0.91	0.85, 0.97	0.007	0.96	0.89, 1.02	0.209	0.90	0.84, 0.96	0.002
	Minority‐language status	1.03	0.96, 1.10	0.469	0.90	0.84, 0.96	0.002	0.97	0.91, 1.04	0.372
	Household composition	0.94	0.87, 1.01	0.071	1.02	0.96, 1.09	0.512	0.91	0.85, 0.97	0.004
	Housing‐transportation	0.97	0.90, 1.04	0.357	0.92	0.86, 0.99	0.018	0.98	0.92, 1.05	0.553
Gastroesophageal junction—White	Total	1.00	0.97, 1.04	0.859	0.97	0.93, 1.00	0.073	0.95	0.92, 0.99	0.013
	Socioeconomic status	1.00	0.97, 1.04	0.806	1.00	0.97, 1.04	0.999	0.97	0.93, 1.00	0.067
	Minority‐language status	0.98	0.94, 1.02	0.256	0.89	0.86, 0.93	< 0.001	0.95	0.92, 0.99	0.007
	Household composition	1.02	0.98, 1.06	0.353	1.06	1.02, 1.10	0.001	0.99	0.95, 1.02	0.487
	Housing‐transportation	1.01	0.97, 1.05	0.634	0.98	0.95, 1.02	0.273	0.97	0.94, 1.01	0.113
Liver—Non‐White	Total	0.88	0.86, 0.91	< 0.001	0.90	0.86, 0.93	< 0.001	1.01	0.98, 1.03	0.575
	Socioeconomic status	0.87	0.85, 0.90	< 0.001	0.94	0.90, 0.98	0.002	1.00	0.98, 1.03	0.735
	Minority‐language status	1.02	1.00, 1.05	0.103	0.82	0.79, 0.86	< 0.001	1.02	0.99, 1.04	0.182
	Household composition	0.86	0.83, 0.88	< 0.001	1.04	1.00, 1.08	0.061	1.02	1.00, 1.04	0.107
	Housing‐transportation	1.01	0.98, 1.04	0.474	0.86	0.82, 0.89	< 0.001	0.99	0.96, 1.01	0.302
Liver—White	Total	0.91	0.89, 0.94	< 0.001	0.89	0.86, 0.92	< 0.001	0.99	0.96, 1.01	0.244
	Socioeconomic status	0.91	0.88, 0.93	< 0.001	0.92	0.89, 0.96	< 0.001	1.0	0.97, 1.02	0.645
	Minority‐language status	1.01	0.98, 1.04	0.597	0.90	0.87, 0.93	< 0.001	1.02	1.00, 1.04	0.086
	Household composition	0.91	0.89, 0.94	< 0.001	1.01	0.97, 1.04	0.732	1.00	0.97, 1.02	0.805
	Housing‐transportation	0.93	0.91, 0.96	< 0.001	0.88	0.85, 0.91	< 0.001	0.97	0.95, 1.00	0.021
Pancreatic head—Non‐White	Total	0.94	0.90, 0.98	0.002	0.92	0.87, 0.96	< 0.001	0.92	0.89, 0.95	< 0.001
	Socioeconomic status	0.92	0.89, 0.96	< 0.001	0.95	0.91, 1.00	0.055	0.93	0.90, 0.96	< 0.001
	Minority‐language status	1.03	0.99, 1.07	0.114	0.87	0.83, 0.91	< 0.001	0.92	0.89, 0.95	< 0.001
	Household composition	0.92	0.88, 0.96	< 0.001	1.05	1.00, 1.10	0.059	0.99	0.96, 1.02	0.563
	Housing‐transportation	1.02	0.98, 1.06	0.308	0.88	0.84, 0.93	< 0.001	0.92	0.89, 0.96	< 0.001
Pancreatic head—White	Total	0.97	0.95, 0.99	0.044	0.97	0.94, 1.00	0.072	0.93	0.90, 0.95	< 0.001
	Socioeconomic status	0.95	0.93, 0.98	< 0.001	1.02	0.99, 1.05	0.196	0.94	0.92, 0.96	< 0.001
	Minority‐language status	1.02	1.00, 1.05	0.114	0.90	0.88, 0.93	< 0.001	0.96	0.94, 0.98	< 0.001
	Household composition	0.96	0.94, 0.99	0.005	1.07	1.04, 1.10	< 0.001	0.96	0.94, 0.98	0.001
	Housing‐transportation	0.99	0.97, 1.02	0.534	0.93	0.90, 0.95	< 0.001	0.92	0.90, 0.94	< 0.001
Pancreatic body and tail—Non‐White	Total	0.93	0.89, 0.98	0.007	0.92	0.85, 0.99	0.041	0.91	0.87, 0.94	< 0.001
	Socioeconomic status	0.89	0.85, 0.94	< 0.001	0.96	0.89, 1.04	0.329	0.92	0.88, 0.96	< 0.001
	Minority‐language status	1.05	1.00, 1.11	0.057	0.88	0.81, 0.95	0.001	0.94	0.90, 0.98	0.007
	Household composition	0.89	0.85, 0.94	< 0.001	1.00	0.93, 1.08	0.938	0.95	0.91, 0.99	0.021
	Housing‐transportation	1.00	0.95, 1.06	0.919	0.90	0.83, 0.97	0.006	0.92	0.88, 0.96	< 0.001
Pancreatic body and tail—White	Total	0.97	0.94, 1.01	0.097	0.95	0.90, 0.99	0.037	0.92	0.90, 0.95	< 0.001
	Socioeconomic status	0.96	0.93, 1.00	0.040	0.99	0.94, 1.04	0.736	0.94	0.91, 0.96	< 0.001
	Minority‐language status	1.04	1.00, 1.07	0.050	0.90	0.85, 0.95	< 0.001	0.96	0.93, 0.99	0.006
	Household composition	0.96	0.92, 0.99	0.011	1.05	1.00, 1.10	0.068	0.95	0.93, 0.98	0.001
	Housing‐transportation	0.97	0.94, 1.01	0.093	0.92	0.88, 0.97	0.002	0.93	0.90, 0.96	< 0.001
Pancreatic, other—Non‐White	Total	0.97	0.90, 1.05	0.419	0.90	0.82, 0.98	0.016	0.89	0.85, 0.93	< 0.001
	Socioeconomic status	0.93	0.86, 1.00	0.054	0.95	0.87, 1.04	0.231	0.92	0.87, 0.96	< 0.001
	Minority‐language status	1.06	0.99, 1.15	0.115	0.86	0.79, 0.94	0.001	0.94	0.90, 0.99	0.016
	Household composition	0.89	0.83, 0.96	0.003	1.03	0.94, 1.13	0.494	0.95	0.90, 0.99	0.018
	Housing‐transportation	1.02	0.95, 1.10	0.576	0.85	0.78, 0.93	0.001	0.92	0.87, 0.96	< 0.001
Pancreatic, other—White	Total	0.96	0.91, 1.01	0.097	0.94	0.88, 0.99	0.034	0.91	0.88, 0.94	< 0.001
	Socioeconomic status	0.95	0.90, 1.00	0.074	0.95	0.90, 1.01	0.123	0.92	0.89, 0.95	< 0.001
	Minority‐language status	1.00	0.95, 1.05	0.975	0.93	0.87, 0.99	0.018	0.97	0.93, 1.00	0.030
	Household composition	0.95	0.90, 1.00	0.044	0.98	0.92, 1.04	0.520	0.93	0.90, 0.96	< 0.001
	Housing‐transportation	0.97	0.92, 1.02	0.218	0.92	0.87, 0.98	0.009	0.95	0.92, 0.98	0.001
Rectum—Non‐White	Total	0.95	0.92, 0.97	< 0.001	1.02	0.99, 1.04	0.222	0.99	0.96, 1.01	0.268
	Socioeconomic status	0.93	0.91, 0.96	< 0.001	1.03	1.00, 1.05	0.029	1.00	0.98, 1.03	0.709
	Minority‐language status	1.00	0.97, 1.03	0.925	0.97	0.94, 0.99	0.007	0.94	0.92, 0.97	< 0.001
	Household composition	0.95	0.93, 0.98	0.001	1.05	1.02, 1.07	< 0.001	1.02	1.00, 1.05	0.057
	Housing‐transportation	0.99	0.96, 1.01	0.314	0.99	0.97, 1.01	0.421	0.97	0.95, 1.0	0.017
Rectum—White	Total	0.97	0.95, 0.99	0.002	0.99	0.98, 1.01	0.519	0.97	0.95, 0.99	< 0.001
	Socioeconomic status	0.99	0.97, 1.01	0.526	1.00	0.99, 1.02	0.809	0.97	0.96, 0.99	0.004
	Minority‐language status	0.93	0.91, 0.95	< 0.001	0.94	0.92, 0.95	< 0.001	0.94	0.93, 0.96	< 0.001
	Household composition	1.02	1.00, 1.04	0.054	1.04	1.03, 1.06	< 0.001	1.02	1.00, 1.04	0.041
	Housing‐transportation	0.97	0.95, 0.98	< 0.001	1.00	0.98, 1.01	0.692	0.98	0.96, 1.0	0.010
Small intestine—Non‐White	Total	0.97	0.90, 1.06	0.537	1.03	0.88, 1.21	0.710	0.91	0.84, 0.99	0.030
	Socioeconomic status	0.96	0.89, 1.05	0.380	1.04	0.88, 1.22	0.654	0.95	0.87, 1.03	0.194
	Minority‐language status	0.95	0.87, 1.03	0.224	0.96	0.81, 1.13	0.600	0.89	0.82, 0.97	0.009
	Household composition	1.00	0.92, 1.09	0.974	1.03	0.87, 1.21	0.745	1.02	0.94, 1.11	0.592
	Housing‐transportation	0.96	0.88, 1.04	0.296	0.97	0.83, 1.15	0.758	0.85	0.78, 0.92	< 0.001
Small intestine—White	Total	0.95	0.89, 1.01	0.116	1.06	0.93, 1.19	0.388	0.93	0.87, 0.99	0.031
	Socioeconomic status	0.97	0.91, 1.03	0.343	0.97	0.86, 1.10	0.663	0.93	0.87, 0.99	0.033
	Minority‐language status	0.96	0.90, 1.02	0.167	1.10	0.97, 1.24	0.129	0.97	0.91, 1.04	0.413
	Household composition	1.02	0.95, 1.08	0.599	1.00	0.89, 1.13	0.961	0.93	0.87, 0.99	0.028
	Housing‐transportation	0.95	0.89, 1.01	0.088	1.12	0.99, 1.26	0.078	0.93	0.87, 0.99	0.018
Stomach—Non‐White	Total	0.95	0.92, 0.98	0.001	0.91	0.87, 0.95	< 0.001	0.92	0.90, 0.95	< 0.001
	Socioeconomic status	0.94	0.91, 0.97	< 0.001	0.93	0.89, 0.96	< 0.001	0.94	0.91, 0.96	< 0.001
	Minority‐language status	1.02	0.99, 1.05	0.125	0.89	0.85, 0.92	< 0.001	0.94	0.92, 0.97	< 0.001
	Household composition	0.94	0.91, 0.97	< 0.001	1.00	0.97, 1.05	0.814	0.97	0.94, 1.00	0.037
	Housing‐transportation	1.00	0.97, 1.03	0.920	0.94	0.91, 0.98	0.005	0.95	0.92, 0.98	< 0.001
Stomach—White	Total	1.00	0.96, 1.04	0.903	0.96	0.91, 1.01	0.118	0.92	0.89, 0.96	< 0.001
	Socioeconomic status	1.00	0.97, 1.04	0.818	1.00	0.95, 1.06	0.877	0.92	0.89, 0.96	< 0.001
	Minority‐language status	1.00	0.97, 1.04	0.881	0.88	0.83, 0.93	< 0.001	0.98	0.94, 1.02	0.256
	Household composition	0.98	0.94, 1.02	0.241	1.07	1.01, 1.13	0.017	0.95	0.92, 0.99	0.009
	Housing‐transportation	1.02	0.98, 1.06	0.253	0.93	0.89, 0.99	0.013	0.95	0.91, 0.98	0.005
Gastrointestinal, other—Non‐White	Total	1.00	0.83, 1.20	0.966	0.76	0.63, 0.90	0.002	0.85	0.77, 0.93	0.001
	Socioeconomic status	1.05	0.87, 1.27	0.583	0.77	0.65, 0.92	0.003	0.88	0.80, 0.97	0.009
	Minority‐language status	0.99	0.82, 1.19	0.895	0.83	0.69, 0.98	0.030	0.90	0.82, 0.99	0.025
	Household composition	1.10	0.91, 1.33	0.314	0.86	0.73, 1.03	0.094	0.93	0.85, 1.02	0.144
	Housing‐transportation	0.85	0.70, 1.02	0.082	0.85	0.71, 1.01	0.059	0.83	0.75, 0.91	< 0.001
Gastrointestinal, other—White	Total	1.04	0.89, 1.22	0.616	0.94	0.83, 1.06	0.285	0.93	0.86, 0.99	0.030
	Socioeconomic status	1.07	0.92, 1.26	0.380	0.93	0.83, 1.05	0.255	0.95	0.88, 1.01	0.122
	Minority‐language status	1.08	0.92, 1.28	0.322	0.93	0.82, 1.05	0.218	0.91	0.85, 0.97	0.007
	Household composition	0.98	0.83, 1.15	0.812	1.02	0.90, 1.15	0.768	1.05	0.98, 1.13	0.166
	Housing‐transportation	1.01	0.86, 1.19	0.868	0.93	0.82, 1.05	0.222	0.89	0.83, 0.96	0.002

^a^ By American Joint Committee on Cancer, 6th Edition (AJCC‐6); Univariate logistic regressions with calculated Odds Ratios (OR) and 95% confidence intervals (95% CI) were performed with lowest relative SVI quintiles set as the reference within each histopathology type.

For surgical resection, Non‐White patients were less likely to receive surgery than White patients with increasing overall social vulnerability for 8 out of 14 GIC sites (largest odds‐ratio difference, gastroesophageal junction [Non‐White, OR 0.92, 95% CI 0.86–0.99; White, OR 1.00, 95% CI 0.97–1.04]) (see Data S1 for all significant sites) (Figure 2A). Specific vulnerabilities in socioeconomic status and household composition contributed the most to these observed associations across both race‐ethnicity cohorts (Table 3).

For radiation therapy, similar trends were observed for 7 out of 14 types (largest odds‐ratio difference, gastrointestinal‐other [Non‐White, OR 0.76, 95% CI 0.63–0.90; White, 0.94, 95% CI 0.83–1.06]) (see Data S1 for all significant sites) (Figure 2B). Specific vulnerabilities in minority‐language status, followed by socioeconomic status and housing‐transportation in magnitude, contributed differentially to these observed trends across both cohorts (Table 3).

In contrast to surgery and radiation therapy, both Non‐White and White cohorts displayed similar magnitudes of decreased chemotherapy receipt with increasing social vulnerability for a majority of GIC sites (see Data S1 for all significant sites) (Figure 2C). Specific vulnerabilities across all themes of socioeconomic status, minority‐language status, household composition, and housing‐transportation contributed differentially to these observed trends (Table 3).

### 3.2 Primary Site‐Specific Trends in Survival Relative to SVI Percentile

**FIGURE 3:**
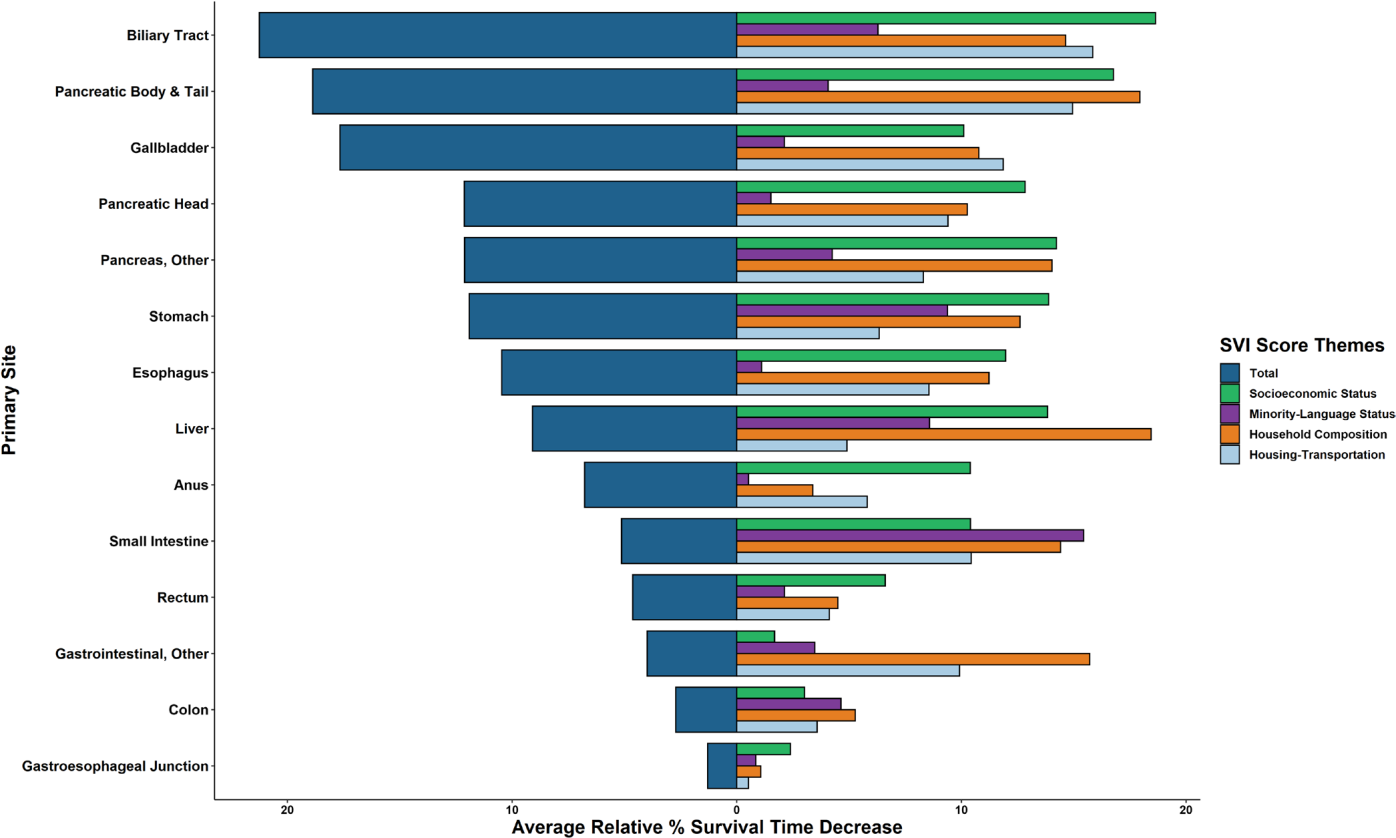
Primary site relative decreases in months survival with increasing SVI Scores. Percentage decreases from lowest to highest‐SVI quintiles based on mean months survived for total‐SVI score and subcomponent SVI‐theme subscores per primary site.

Social vulnerability was strongly associated with survival differences among most patients with GIC (Figure S2). Survival among patients residing in the lowest versus highest SVI county had differences in survival that ranged from as high as a 21.3% decrease (6.85–5.40 months) among patients with biliary tract cancer to as low as a 1.3% decrease (8.48–8.37 months) among individuals with a gastroesophageal junction cancer (Figure 3). The impact of social vulnerability on survival varied across GIC diagnoses, having the most pronounced effect on patients with cancers of esophagus, stomach, liver, pancreas, and biliary tract (all *p* < 0.001) (Figure S5). Of note, social vulnerability subthemes that contributed the most to differences in survival were socioeconomic status and household composition, followed by housing‐transportation, and then minority‐language status (Figure 3).

**FIGURE 4:**
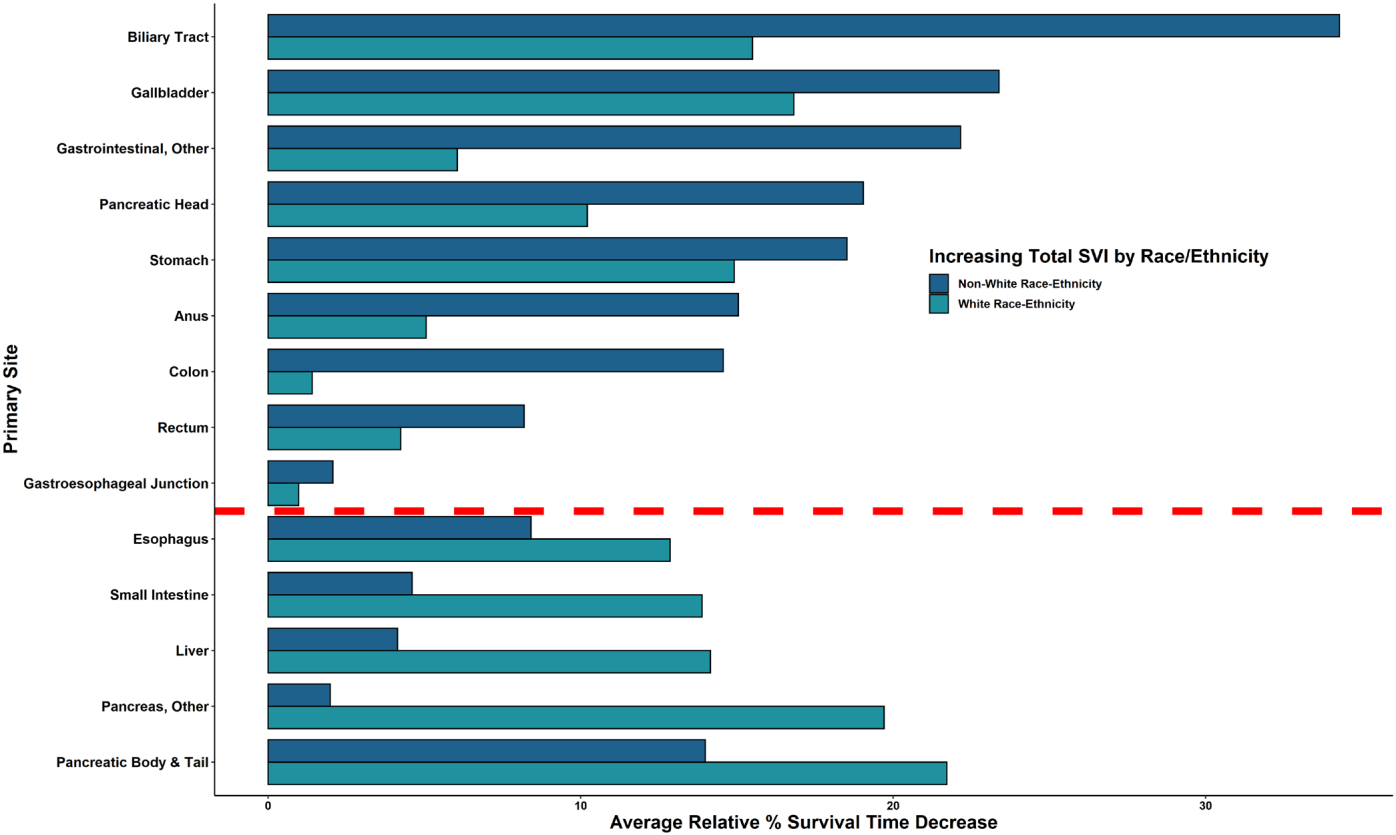
Race/ethnicity‐comparison of relative decreases in months survival with increasing total SVI Scores across primary sites. Percentage decreases from lowest to highest‐SVI quintiles based on mean months survived for total‐SVI score and subcomponent SVI‐theme subscores per primary site.

When differentiating the influence of increasing social vulnerability with survival among White and Non‐White patients diagnosed with GIC, both cohorts displayed associated decreases in survival (Figures S3 and S4). However, Non‐White patients experienced higher decreases in survival period compared with White patients for 9/14 GIC types analyzed, with the maximum difference of 18.78% in biliary tract (Non‐White, 34.29% decrease [7.91–5.20 months]; White, 15.50% decrease [6.66–5.63 months]) and minimum difference of 1.1% in gastroesophageal junction (Non‐White, 2.07% decrease [8.51–8.33 months]; White, 0.98% decrease [8.47–8.39 months]). White patients experienced higher decreases in survival period versus non‐White patients for 5/14 GIC types analyzed. The maximum difference was 17.7% for pancreas—other (White, 19.71% decrease [4.48–3.59 months]; Non‐White, 1.99% decrease [4.31–4.22 months]), and the minimum difference was 4.45% for esophagus (White, 12.86% decrease [8.13–7.09 months]; Non‐White, 8.41% decrease [7.31–6.69 months]) (Figure 4). For White patients with GIC, specific vulnerabilities in housing‐transportation and household composition, followed by socioeconomic status, contributed the most to these observed overall vulnerability trends (Figure S6). For Non‐White patients with GIC, minority‐language status, housing transportation, and household composition contributed similarly to observed overall vulnerability trends (Figure S7).

## 4 Discussion

This nationally representative cohort of GIC patients serves to highlight how decreases in systemic and surgical therapy receipt and survival period were differentially associate with increasing overall social vulnerability and specific types of social vulnerability. Moreover, these differences were exacerbated relative to treatment receipt and survival period among Non‐White patients with GIC versus White patients. The current study was important as the data provide information that identified which social vulnerability factors specifically impact non‐surgical treatment disparities across a majority of GIC types, as well as demonstrated surgical treatment disparities among patients with understudied GIC types (e.g., non‐hepatopancreatobiliary cancers). Furthermore, we demonstrated a wider variety of SDoH‐factors to contextualize GIC disparities relative to long‐term surveillance and survival/mortality that prior literature had not fully elucidated.

To our knowledge, this study principally demonstrated varied social vulnerability‐related associations with GIC disparities relative to chemotherapy and radiotherapy receipt in a large, national scale dataset many GIC types. While racial disparities have been observed in adjuvant therapy receipt for certain GICs, data from this current study examined a broader cohort of GIC with consideration of other SDoH factors beyond race‐ethnicity having a dramatic impact on outcomes [7, 12, 19]. Specifically, we confirmed how SDoH‐vulnerabilities beyond race/ethnicity had greater impact. For instance, housing‐transportation type & access, a notable SDoH‐theme has been strongly linked to worse access to quality surgical care at high‐volume surgical centers [20]. In the current study, housing‐transportation had a larger magnitude‐associations with decreased receipt of chemotherapy receipt than race‐ethnicity and English‐language proficiency. Furthermore, socioeconomic vulnerabilities had comparable association with worse outcomes as minoritized race‐ethnicity across overall trends of poor chemotherapy receipt, which were consistent with previous work that noted an intersection of socioeconomic status and race/ethnicity [3, 11]. A similar trend was noted for receipt of radiation therapy for 7 out of 14 types with specific vulnerabilities related to minority‐language status, followed by socioeconomic status and housing‐transportation in magnitude. Collectively, SDoH‐factors had a marked impact on the lack of utilization of non‐operative management of several GIC at the national level [21, 22, 23]. In turn, the data emphasize how social factors beyond race‐ethnicity impact receipt and utilization of non‐operative cancer therapies for patients with GIC.

Notably, increased social vulnerability was associated with decreased utilization of surgical resection across 8/14 GIC types. Disparities in surgical utilization for GICs including esophageal, hepatic, pancreas, and biliary tract cancers were noted, and were directly impacted by differences related to socioeconomic status, race‐ethnicity, and geography‐transportation. Of note, many of the GIC types analyzed were characterized by the lowest‐magnitude/highest‐impact associations relative to HT‐ or SES‐vulnerability, both of which were lower magnitude/higher impact than ML‐vulnerabilities. Data on these themes and how each was differentially associated with variations in surgical treatment utilization across GICs further contextualized the relationship between socioeconomic‐status and race‐ethnicity [3, 11]. Prior investigations of the surgical resection trends among other oncologic pathologies have showcased similar trends of SES‐ and HT‐vulnerabilities playing substantial roles in conferring this detrimental association, such as those of the head–neck, endocrine, skin, and central nervous systems [17, 18, 24, 25, 26, 27, 28, 29, 30, 31, 32]. Given calls to address systemic social biases that exacerbate racial differences in GIC treatment, data from the current study specifically highlights how SES and HT‐based differences may represent important policy targets of policy to mitigate disparities in care across diverse sociodemographic/racial‐ethnic groups [33].

The current study specifically highlights which subthemes of social vulnerability contributed the most to overall and individual racial/ethnic differences in the treatment of GIC. As overall social vulnerability increased, decreased utilization of surgical resection and radiation therapy receipt were more pronounced among Non‐White GIC patients versus White GIC patients. Here, Non‐White patients experienced worse disparities in surgery and radiation therapy than White patients that not only corroborated prior findings [6, 10, 34], but also went beyond them by specifically quantifying how race/ethnic disparity differences are delineated across specific community‐level social vulnerabilities. Specifically, ML‐vulnerability of the community more strongly influenced disparities in radiation therapy receipt versus other SDoH factors, suggesting that the racial‐ethnic makeup and language capacity inherent in a Non‐White GIC‐patient community needs to be addressed to improve care of these individuals.

Alongside this influence, SES‐vulnerability also showed substantial impact in effecting poor multi‐modal treatment receipt for non‐white patients compared to white patients. Although race‐ethnicity has been examined relative to SES among patients undergoing surgery [35, 36], the impact and interaction of race/ethnicity and SDoH on non‐operative GIC therapy such as chemotherapy and radiation therapy has not been studied until this present study. Furthermore, lesser‐studied SDoH such as housing‐transportation type and household composition were also identified as important drivers of multi‐modality care of patients with GIC, indicating that multifaceted social vulnerability impacted not only surgery but also receipt of radiation therapy receipt among both White and Non‐White GIC patients.

Given these vast differences in treatment receipt, this study also analyzed the downstream effect of social vulnerability on worse overall survival among many patients GIC. In fact, the impact on survival ranged from as high as a 21% decrease in survival length across observed vulnerability trends, with an increased detrimental effect on survival observed among Non‐White GIC‐patients with a majority of GIC versus White individuals. Compared to prior literature, these trends of downstream multi‐modal treatment effects were not only present within solid tumor‐based SVI studies, such as ours and the ones prior referenced, but also in hematologic cancer types ranging upwards of over 50% survival differences [37, 38].

Since the pandemic, social vulnerability has been a focus of public health initiatives, (e.g., hundreds of COVID‐19 test based in areas of high social vulnerability) [39]. Within GIC, adoption of similar outreach efforts are needed to implement policies aimed at addressing some of the SDoH subthemes identified in the current study (i.e., transportation, language barriers, economic mobility, etc.). For instance, publicly led initiatives, such as the CDC‐sponsored tobacco cessation programs in Mississippi or accessible testing programs in New York City [40], have utilized information derived from large‐data analyses to allocate resources. As national support for cancer equity grows [41], public health efforts and investigations, such as increased low‐cost screening availability in socially vulnerable areas or the provision of supportive care services of transportation and fee waivers for vulnerable GIC patients, should continue to derive thoughtful action based on innovative, large‐data investigations to understand and address the multifaceted impact of SDoH in GIC and the broader oncologic field.

Despite the strengths of this study, there were several limitations. The SEER database did not encompass variables to characterize details on operation and systemic treatment (e.g., sequence), including the lack of data on immunotherapies. In addition, most of the study population was White, which may have skewed the results. Although constituted by 15 SDoH‐factors, the CDC‐SVI did not measure all SDoH that would be of clinical interest, as more detailed measures cannot be ascertained due to the design of CDC‐SVI.

## Author Contributions

**David J. Fei‐Zhang:** conceptualization (lead), data curation (lead), formal analysis (lead), funding acquisition (lead), investigation (lead), methodology (lead), project administration (lead), resources (lead), software (lead), validation (lead), visualization (lead), writing – original draft (lead), writing – review and editing (lead). **David J. Bentrem:** conceptualization (equal), investigation (equal), methodology (equal), project administration (equal), resources (equal), supervision (supporting), validation (equal), visualization (equal), writing – review and editing (equal). **Jeffrey D. Wayne:** conceptualization (equal), data curation (equal), investigation (equal), methodology (equal), project administration (equal), resources (equal), supervision (supporting), validation (equal), visualization (equal), writing – review and editing (equal). **Lifang Hou:** conceptualization (supporting), investigation (equal), methodology (equal), project administration (supporting), resources (supporting), supervision (supporting), visualization (equal), writing – review and editing (supporting). **Peiwen Fei:** conceptualization (equal), data curation (supporting), funding acquisition (supporting), investigation (equal), methodology (equal), project administration (equal), resources (equal), software (supporting), supervision (equal), validation (supporting), visualization (supporting), writing – original draft (equal), writing – review and editing (equal). **Timothy M. Pawlik:** conceptualization (equal), data curation (equal), formal analysis (equal), funding acquisition (equal), investigation (equal), methodology (equal), project administration (equal), supervision (equal), validation (equal), visualization (equal), writing – original draft (equal), writing – review and editing (equal).

## Conflicts of Interest

The authors declare no conflicts of interest.

## Disclaimer

The content is solely the responsibility of the authors and does not necessarily represent the official views of the Centers for Disease Control and Prevention or the National Cancer Institute.

## Supporting information


**Figure S1.** Schematic Workflow of SVI and SEER Database Manipulation.

**Figure S2.** Kaplan–Meier Survival Analyses of Months‐Survival Trends with Increasing SVI. Differences in survival between the lowest and highest relative SVI quintiles were assessed for the overall gastrointestinal cancer cohort. Log‐rank testing was conducted for assessing statistical significance.

**Figure S3.** Kaplan–Meier Survival Analyses of Months‐Survival Trends with Increasing SVI for White Race/Ethnicity Patients. Differences in survival between the lowest and highest relative SVI quintiles were assessed for the White race/ethnicity gastrointestinal cancer cohort. Log‐rank testing was conducted for assessing statistical significance.

**Figure S4.** Kaplan–Meier Survival Analyses of Months‐Survival Trends with Increasing SVI for Non‐White Race/Ethnicity Patients. Differences in survival between the lowest and highest relative SVI quintiles were assessed for the Non‐White race/ethnicity gastrointestinal cancer cohort. Log‐rank testing was conducted for assessing statistical significance.

**Figure S5.** Linear Regression of Months‐Survival Trends with Increasing SVI Scores. Primary site‐classified diagnoses were split into quintiles of (A) total SVI, (B) socioeconomic status, (C) minority‐language, (D) household‐composition, and (E) housing‐transport and assessed by linear regression across quintiles for significance.

**Figure S6.** Primary Site Relative Decreases in Months Survival with Increasing SVI Scores for White Race/Ethnicity Patients. Percentage decreases from lowest to highest‐SVI quintiles based on mean months survived for total‐SVI score and subcomponent SVI‐theme subscores per primary site for self‐identified, non‐Hispanic White race/ethnicity GIC patients.

**Figure S7.** Primary Site Relative Decreases in Months Survival with Increasing SVI Scores for Non‐White Race/Ethnicity Patients. Percentage decreases from lowest to highest‐SVI quintiles based on mean months survived for total‐SVI score and subcomponent SVI‐theme subscores per primary site for self‐identified non‐White race/ethnicity GIC patients.

**Table S1.** Patient Characteristics by Socioeconomic Status SVI Score.

**Table S2.** Patient Characteristics by Minority‐Language Status SVI Score.

**Table S3.** Patient Characteristics by Household Composition Status SVI Score.

**Table S4.** Patient Characteristics by Housing‐Transportation Status SVI Score.

Data S1.

## Data Availability

All authors had full access to all the data in the study and take full responsibility for the integrity of the data and the accuracy of the data analysis. These data and their defined variables are publicly available through the SEER administrators (https://seer.cancer.gov/index.html) and the CDC‐SVI website (https://www.atsdr.cdc.gov/placeandhealth/).
