# Supplementary figures and images for: Associations of Social Vulnerability and Race‐Ethnicity With Gastrointestinal Cancers in the United States
Source: Cancer Med. 2025 Mar 5;14(5):e70591. doi: 10.1002/cam4.70591 (PMC11880827; doi:10.1002/cam4.70591)

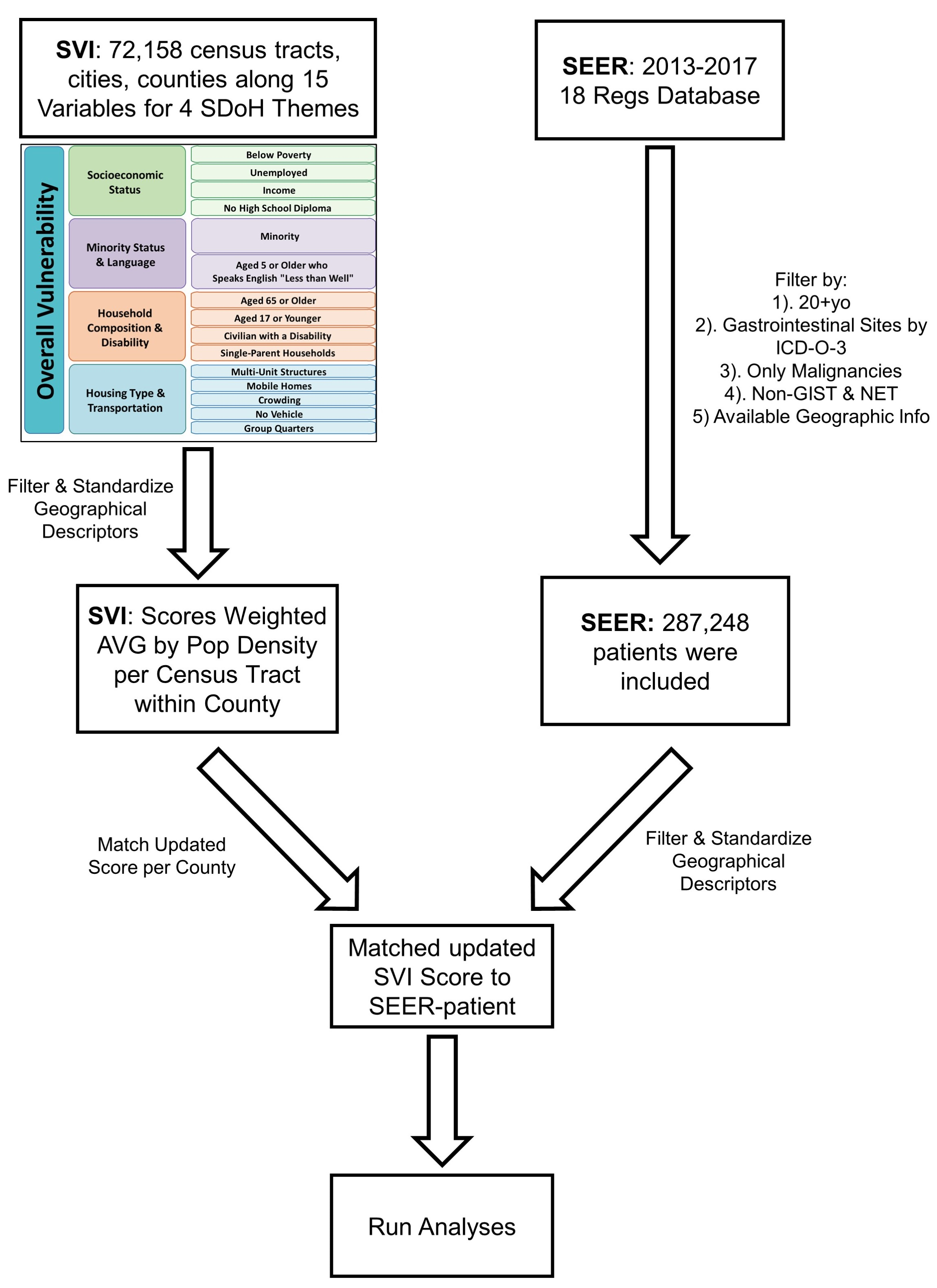

Supplement: Supplementary file 1 — Figure S1. Schematic Workflow of SVI and SEER Database Manipulation. [file CAM4-14-e70591-s002.tiff]

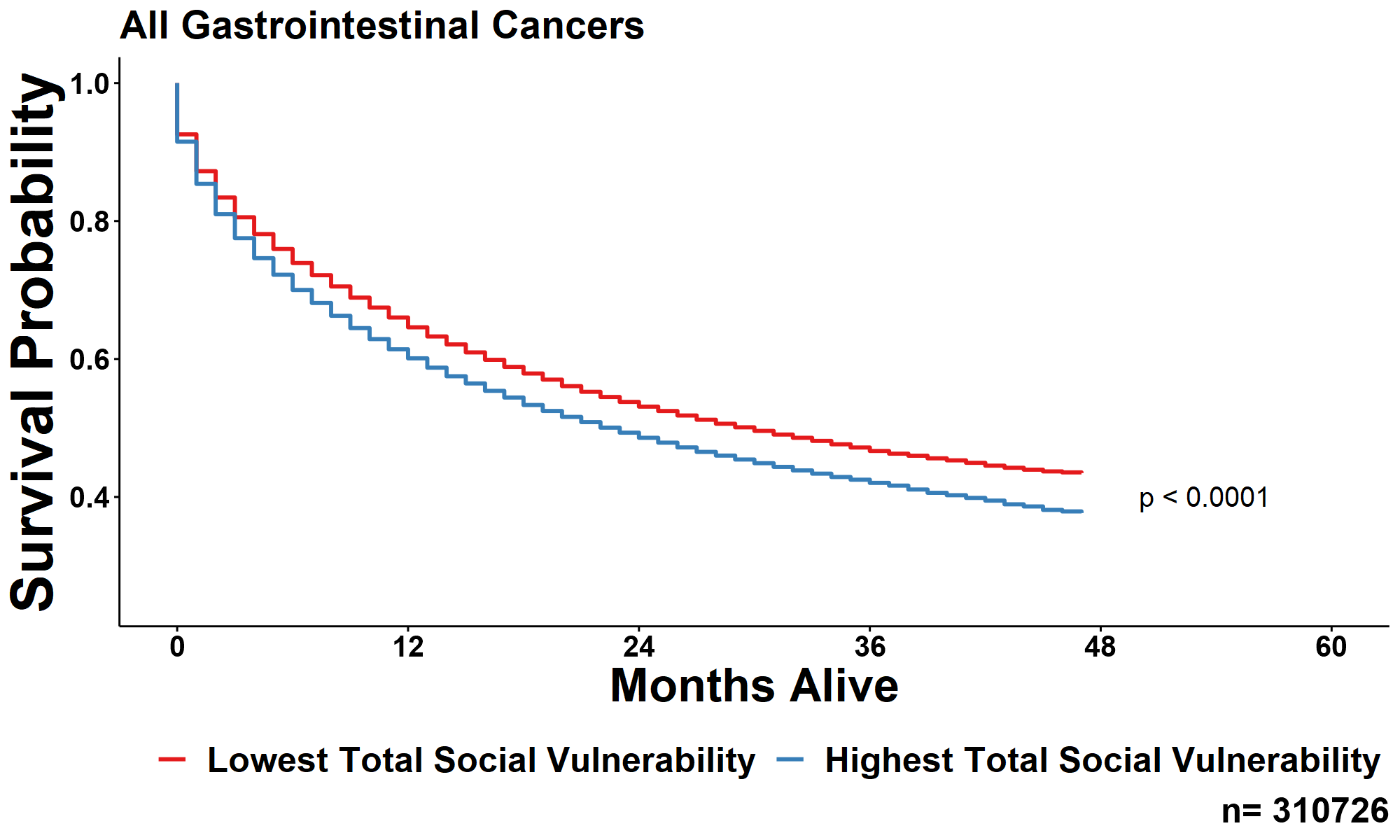

Supplement: Supplementary file 2 — Figure S2. Kaplan–Meier Survival Analyses of Months‐Survival Trends with Increasing SVI. Differences in survival between the lowest and highest relative SVI quintiles were assessed for the overall gastrointestinal cancer cohort. Log‐rank testing was conducted for assessing statistical significance. [file CAM4-14-e70591-s012.tiff]

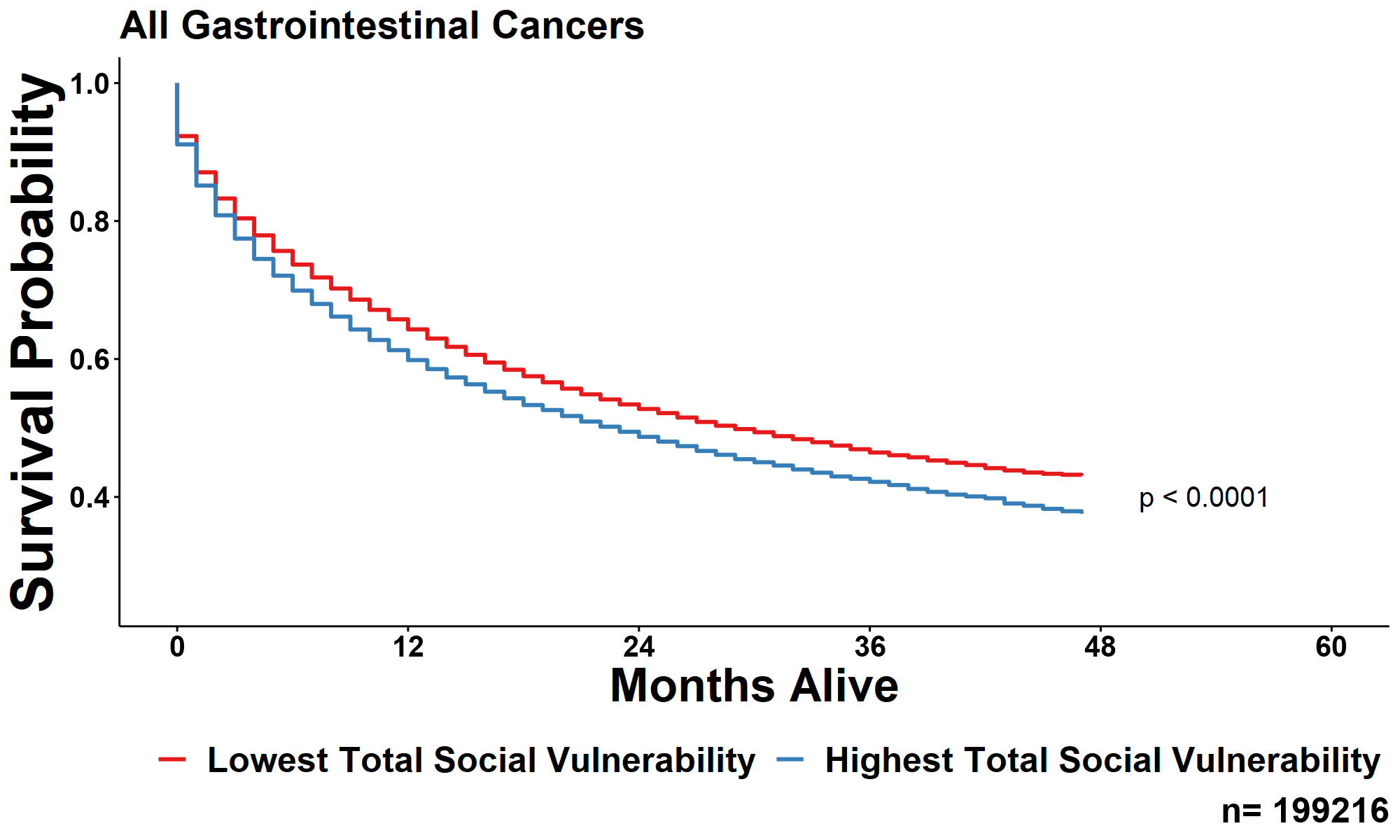

Supplement: Supplementary file 3 — Figure S3. Kaplan–Meier Survival Analyses of Months‐Survival Trends with Increasing SVI for White Race/Ethnicity Patients. Differences in survival between the lowest and highest relative SVI quintiles were assessed for the White race/ethnicity gastrointestinal cancer cohort. Log‐rank testing was conducted for assessing statistical significance. [file CAM4-14-e70591-s005.tiff]

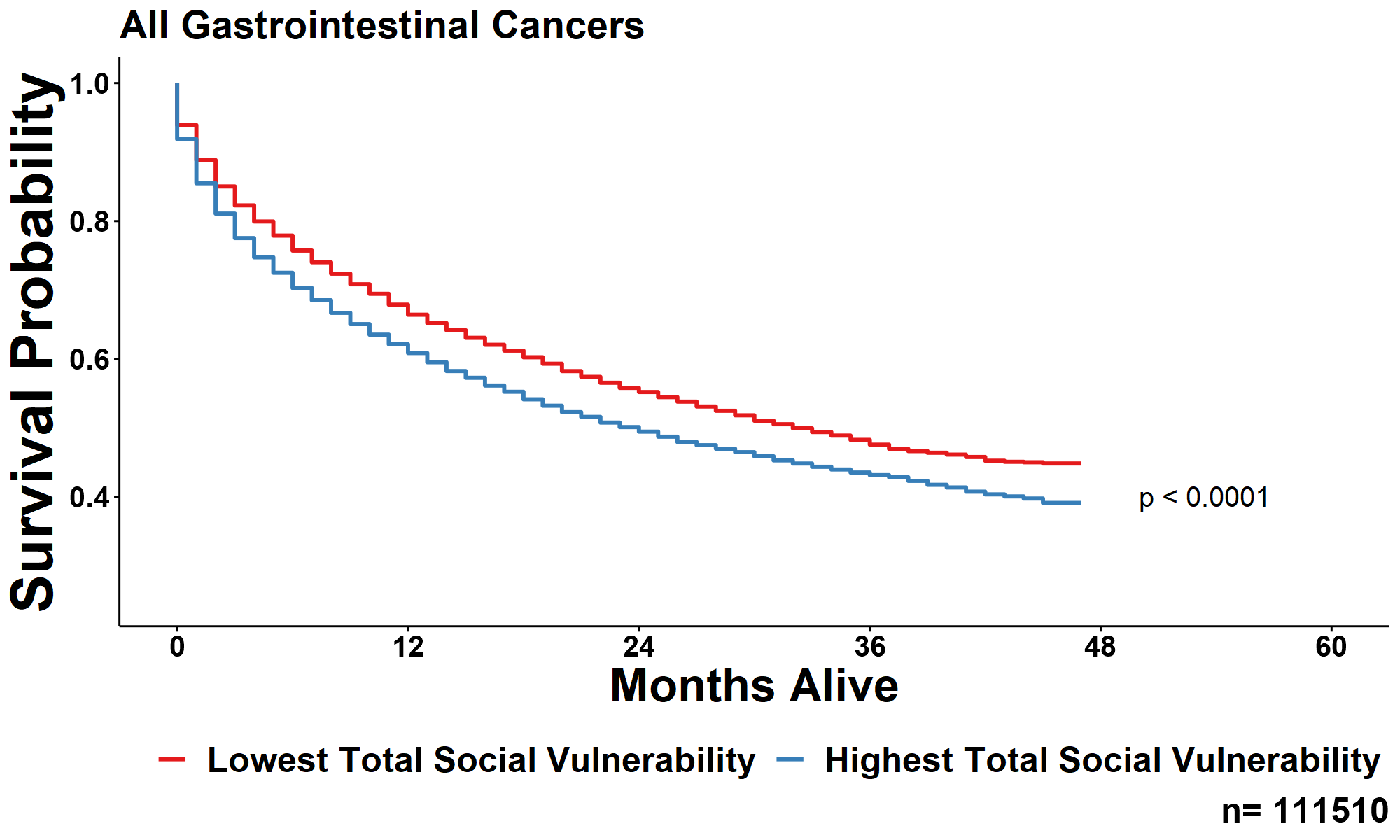

Supplement: Supplementary file 4 — Figure S4. Kaplan–Meier Survival Analyses of Months‐Survival Trends with Increasing SVI for Non‐White Race/Ethnicity Patients. Differences in survival between the lowest and highest relative SVI quintiles were assessed for the Non‐White race/ethnicity gastrointestinal cancer cohort. Log‐rank testing was conducted for assessing statistical significance. [file CAM4-14-e70591-s008.tiff]

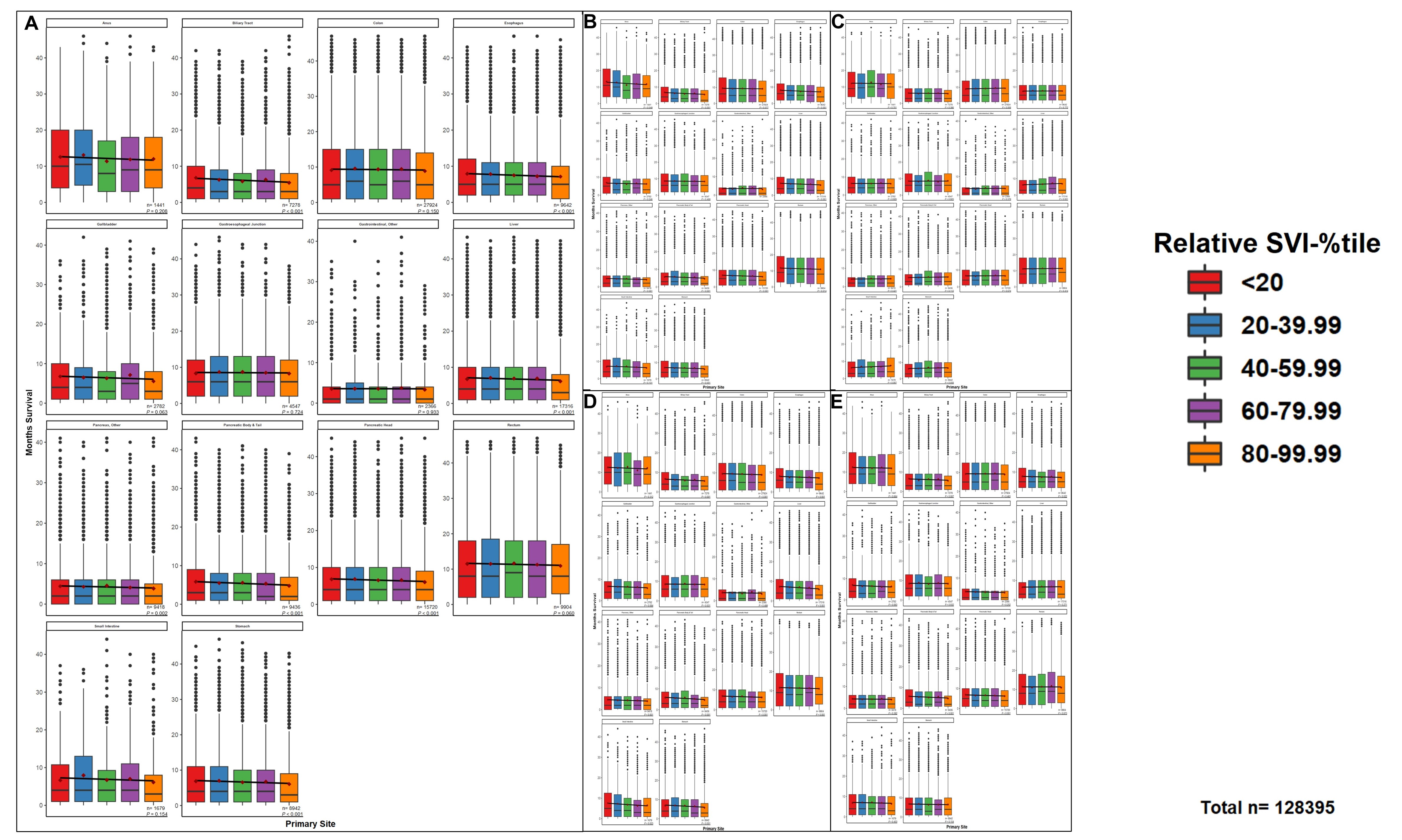

Supplement: Supplementary file 5 — Figure S5. Linear Regression of Months‐Survival Trends with Increasing SVI Scores. Primary site‐classified diagnoses were split into quintiles of (A) total SVI, (B) socioeconomic status, (C) minority‐language, (D) household‐composition, and (E) housing‐transport and assessed by linear regression across quintiles for significance. [file CAM4-14-e70591-s003.tiff]

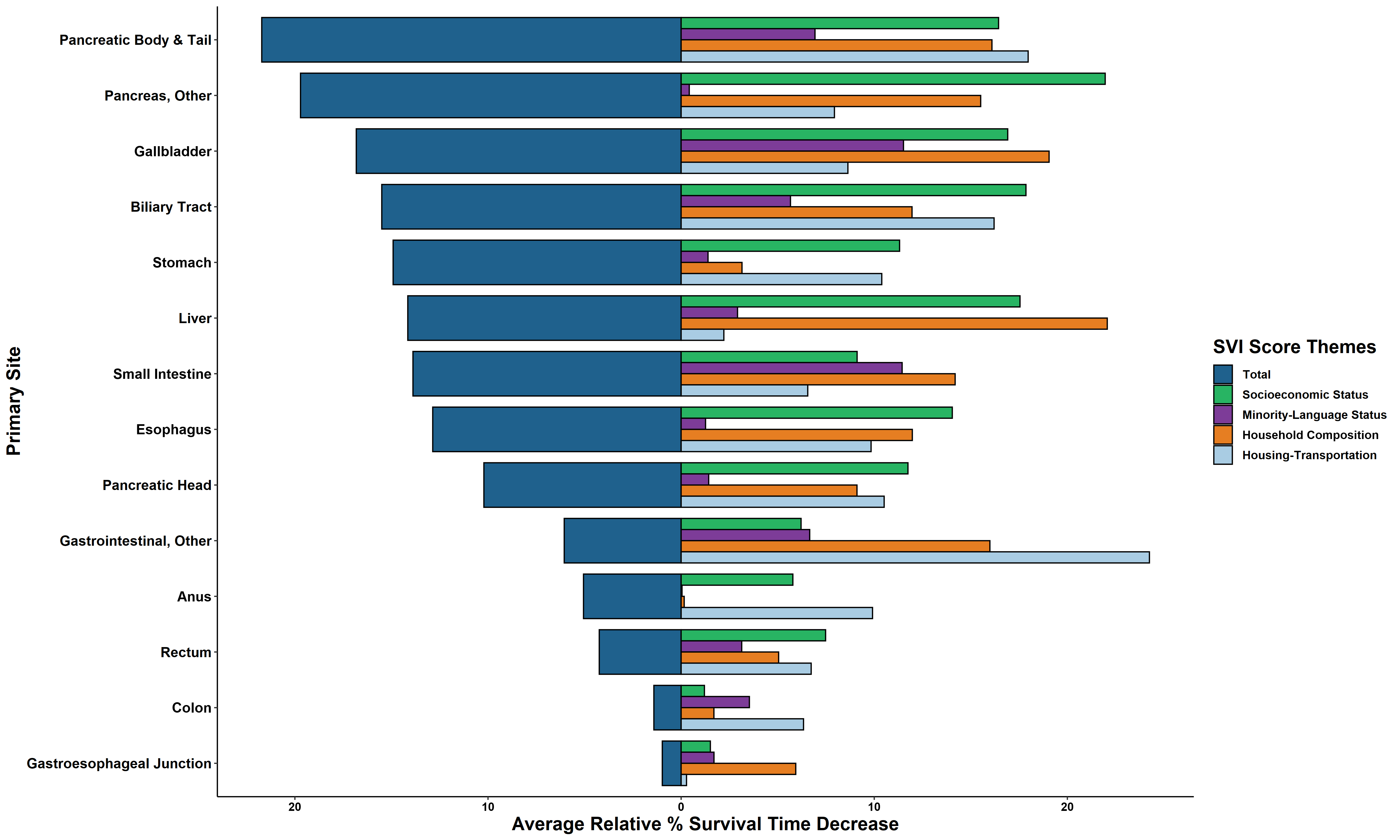

Supplement: Supplementary file 6 — Figure S6. Primary Site Relative Decreases in Months Survival with Increasing SVI Scores for White Race/Ethnicity Patients. Percentage decreases from lowest to highest‐SVI quintiles based on mean months survived for total‐SVI score and subcomponent SVI‐theme subscores per primary site for self‐identified, non‐Hispanic White race/ethnicity GIC patients. [file CAM4-14-e70591-s010.tiff]

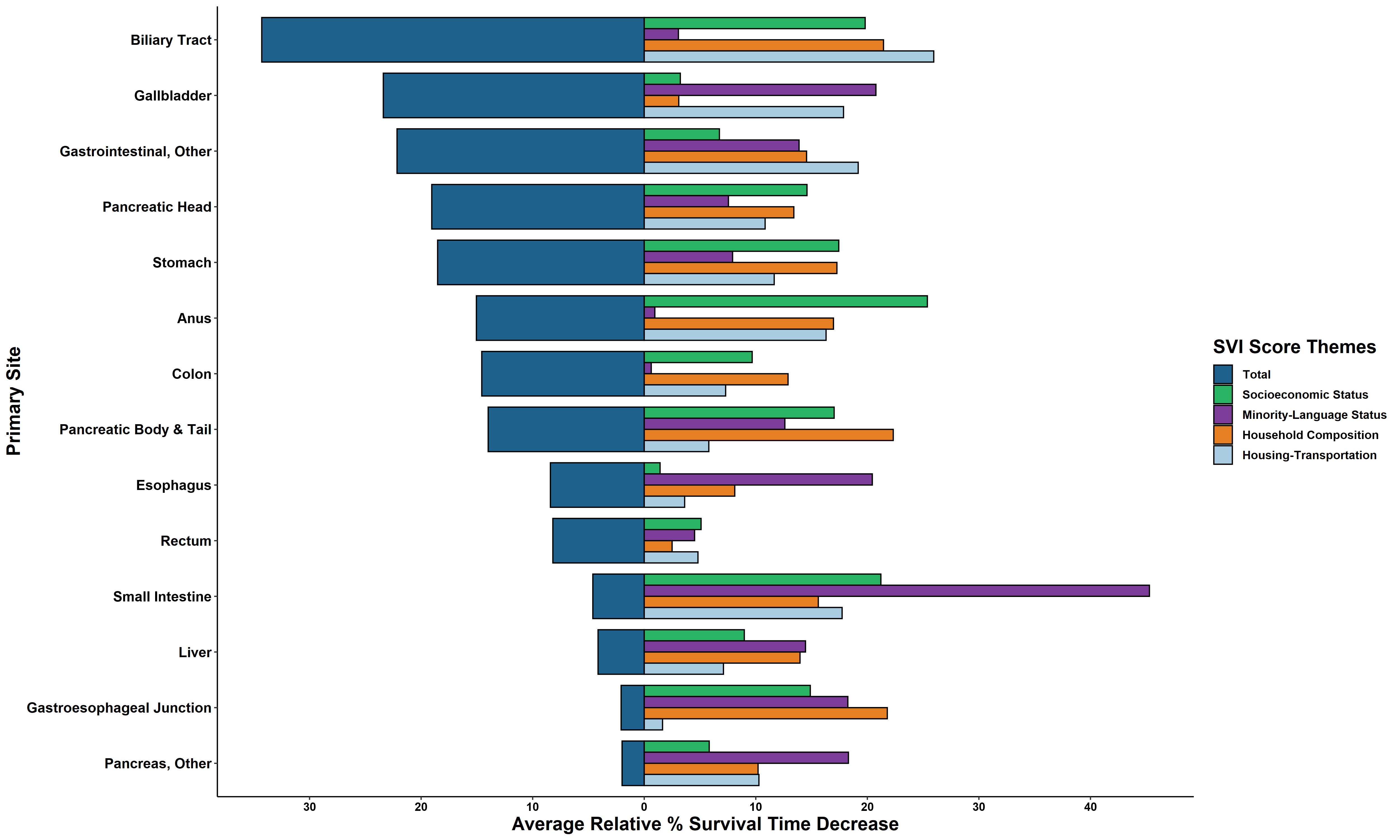

Supplement: Supplementary file 7 — Figure S7. Primary Site Relative Decreases in Months Survival with Increasing SVI Scores for Non‐White Race/Ethnicity Patients. Percentage decreases from lowest to highest‐SVI quintiles based on mean months survived for total‐SVI score and subcomponent SVI‐theme subscores per primary site for self‐identified non‐White race/ethnicity GIC patients. [file CAM4-14-e70591-s006.tiff]
