# Supplementary material for: Associations of Social Vulnerability and Race‐Ethnicity With Gastrointestinal Cancers in the United States
Source: Cancer Med. 2025 Mar 5;14(5):e70591. doi: 10.1002/cam4.70591 (PMC11880827; doi:10.1002/cam4.70591)
Supplement: Supplementary file 8 — Table S1. Patient Characteristics by Socioeconomic Status SVI Score. [file CAM4-14-e70591-s007.docx]

|  | **Socioeconomic SVI Subscore** | | | | | |  |
| --- | --- | --- | --- | --- | --- | --- | --- |
| **Characteristic** | **Overall**, N = 287248 (100%) | **0.000-0.199**, N = 5243 (1.8%) | **0.200-0.399**, N = 78742 (27%) | **0.400-0.599**, N = 132355 (46%) | **0.600-0.799**, N = 63063 (22%) | **0.800-0.999**, N = 7845 (2.7%) | **p-value** |
| **Age** |  |  |  |  |  |  | <0.001 |
| 20-44 years | 12,110 (4.2%) | 185 (3.5%) | 3,280 (4.2%) | 5,661 (4.3%) | 2,663 (4.2%) | 321 (4.1%) |  |
| 45-64 years | 105,661 (37%) | 1,779 (34%) | 27,659 (35%) | 48,298 (36%) | 24,756 (39%) | 3,169 (40%) |  |
| 65-84 years | 137,674 (48%) | 2,558 (49%) | 37,890 (48%) | 63,505 (48%) | 30,008 (48%) | 3,713 (47%) |  |
| 85+ years | 31,803 (11%) | 721 (14%) | 9,913 (13%) | 14,891 (11%) | 5,636 (8.9%) | 642 (8.2%) |  |
| **Sex** |  |  |  |  |  |  | <0.001 |
| Male | 162,387 (57%) | 2,892 (55%) | 44,292 (56%) | 74,217 (56%) | 36,409 (58%) | 4,577 (58%) |  |
| Female | 124,861 (43%) | 2,351 (45%) | 34,450 (44%) | 58,138 (44%) | 26,654 (42%) | 3,268 (42%) |  |
| **Race** |  |  |  |  |  |  | <0.001 |
| White | 185,450 (65%) | 4,309 (82%) | 54,575 (69%) | 81,599 (62%) | 39,396 (62%) | 5,571 (71%) |  |
| Hispanic | 37,956 (13%) | 437 (8.3%) | 6,180 (7.8%) | 21,520 (16%) | 8,983 (14%) | 836 (11%) |  |
| Black | 34,239 (12%) | 181 (3.5%) | 5,516 (7.0%) | 15,648 (12%) | 11,768 (19%) | 1,126 (14%) |  |
| Asian or Pacific Islander | 26,267 (9.1%) | 250 (4.8%) | 11,713 (15%) | 12,145 (9.2%) | 2,081 (3.3%) | 78 (1.0%) |  |
| Native American | 1,866 (0.6%) | 38 (0.7%) | 331 (0.4%) | 694 (0.5%) | 588 (0.9%) | 215 (2.7%) |  |
| Unknown | 1,470 (0.5%) | 28 (0.5%) | 427 (0.5%) | 749 (0.6%) | 247 (0.4%) | 19 (0.2%) |  |
| **Region** |  |  |  |  |  |  | <0.001 |
| Midwest | 26,674 (9.3%) | 343 (6.5%) | 11,634 (15%) | 7,338 (5.5%) | 7,359 (12%) | 0 (0%) |  |
| Northeast | 45,747 (16%) | 3,256 (62%) | 21,883 (28%) | 19,981 (15%) | 627 (1.0%) | 0 (0%) |  |
| South | 66,701 (23%) | 323 (6.2%) | 4,455 (5.7%) | 29,773 (22%) | 26,023 (41%) | 6,127 (78%) |  |
| West | 148,126 (52%) | 1,321 (25%) | 40,770 (52%) | 75,263 (57%) | 29,054 (46%) | 1,718 (22%) |  |
| **Primary Site** |  |  |  |  |  |  | <0.001 |
| Anus | 7,274 (2.5%) | 144 (2.7%) | 1,963 (2.5%) | 3,418 (2.6%) | 1,587 (2.5%) | 162 (2.1%) |  |
| Biliary Tract | 10,510 (3.7%) | 225 (4.3%) | 3,059 (3.9%) | 4,956 (3.7%) | 2,007 (3.2%) | 263 (3.4%) |  |
| Colon | 97,990 (34%) | 1,820 (35%) | 26,491 (34%) | 44,651 (34%) | 22,048 (35%) | 2,980 (38%) |  |
| Esophagus | 16,276 (5.7%) | 310 (5.9%) | 4,629 (5.9%) | 7,200 (5.4%) | 3,690 (5.9%) | 447 (5.7%) |  |
| Gallbladder | 4,550 (1.6%) | 74 (1.4%) | 1,204 (1.5%) | 2,237 (1.7%) | 918 (1.5%) | 117 (1.5%) |  |
| Gastroesophageal Junction | 7,961 (2.8%) | 147 (2.8%) | 2,412 (3.1%) | 3,627 (2.7%) | 1,580 (2.5%) | 195 (2.5%) |  |
| Gastrointestinal, Other | 3,097 (1.1%) | 58 (1.1%) | 840 (1.1%) | 1,482 (1.1%) | 645 (1.0%) | 72 (0.9%) |  |
| Liver | 31,105 (11%) | 423 (8.1%) | 8,074 (10%) | 14,671 (11%) | 7,239 (11%) | 698 (8.9%) |  |
| Pancreas, Other | 13,369 (4.7%) | 287 (5.5%) | 3,725 (4.7%) | 6,163 (4.7%) | 2,869 (4.5%) | 325 (4.1%) |  |
| Pancreatic Body & Tail | 13,860 (4.8%) | 299 (5.7%) | 4,154 (5.3%) | 6,298 (4.8%) | 2,799 (4.4%) | 310 (4.0%) |  |
| Pancreatic Head | 22,569 (7.9%) | 432 (8.2%) | 6,410 (8.1%) | 10,389 (7.8%) | 4,765 (7.6%) | 573 (7.3%) |  |
| Rectum | 40,351 (14%) | 743 (14%) | 10,927 (14%) | 18,256 (14%) | 9,163 (15%) | 1,262 (16%) |  |
| Small Intestine | 3,075 (1.1%) | 62 (1.2%) | 894 (1.1%) | 1,426 (1.1%) | 627 (1.0%) | 66 (0.8%) |  |
| Stomach | 15,261 (5.3%) | 219 (4.2%) | 3,960 (5.0%) | 7,581 (5.7%) | 3,126 (5.0%) | 375 (4.8%) |  |
| **TNM/AJCC Combined Stage** |  |  |  |  |  |  | 0.225 |
| Stage I-III | 175,818 (67%) | 3,175 (67%) | 48,992 (67%) | 80,325 (67%) | 38,545 (67%) | 4,781 (67%) |  |
| Stage IV & Above | 86,936 (33%) | 1,595 (33%) | 23,849 (33%) | 39,920 (33%) | 19,210 (33%) | 2,362 (33%) |  |
| **Primary Surgery Performed** |  |  |  |  |  |  | <0.001 |
| No Surgery | 130,562 (47%) | 2,388 (47%) | 35,580 (47%) | 60,222 (47%) | 29,059 (48%) | 3,313 (45%) |  |
| Surgery | 146,487 (53%) | 2,667 (53%) | 40,929 (53%) | 67,162 (53%) | 31,639 (52%) | 4,090 (55%) |  |
| **Radiation Therapy Performed** |  |  |  |  |  |  | <0.001 |
| No Therapy | 237,145 (83%) | 4,332 (83%) | 64,397 (82%) | 110,197 (83%) | 51,818 (82%) | 6,401 (82%) |  |
| Therapy | 50,103 (17%) | 911 (17%) | 14,345 (18%) | 22,158 (17%) | 11,245 (18%) | 1,444 (18%) |  |
| **Chemotherapy Performed** |  |  |  |  |  |  | <0.001 |
| No Therapy | 163,426 (57%) | 2,769 (53%) | 43,501 (55%) | 75,857 (57%) | 36,833 (58%) | 4,466 (57%) |  |
| Therapy | 123,822 (43%) | 2,474 (47%) | 35,241 (45%) | 56,498 (43%) | 26,230 (42%) | 3,379 (43%) |  |
| **Vital Status on Last Follow-up** |  |  |  |  |  |  | <0.001 |
| Alive | 153,472 (53%) | 2,984 (57%) | 43,611 (55%) | 70,657 (53%) | 32,217 (51%) | 4,003 (51%) |  |
| Dead | 133,776 (47%) | 2,259 (43%) | 35,131 (45%) | 61,698 (47%) | 30,846 (49%) | 3,842 (49%) |  |
