# Supplementary material for: Associations of Social Vulnerability and Race‐Ethnicity With Gastrointestinal Cancers in the United States
Source: Cancer Med. 2025 Mar 5;14(5):e70591. doi: 10.1002/cam4.70591 (PMC11880827; doi:10.1002/cam4.70591)
Supplement: Supplementary file 9 — Table S2. Patient Characteristics by Minority‐Language Status SVI Score. [file CAM4-14-e70591-s009.docx]

|  | **Minority-Language SVI Subscore** | | | | | |  |
| --- | --- | --- | --- | --- | --- | --- | --- |
| **Characteristic** | **Overall**, N = 287248 (100%) | **0.000-0.199**, N = 13718 (4.8%) | **0.200-0.399**, N = 36543 (13%) | **0.400-0.599**, N = 82793 (29%) | **0.600-0.799**, N = 96522 (34%) | **0.800-0.999**, N = 57672 (20%) | **p-value** |
| **Age** |  |  |  |  |  |  | <0.001 |
| 20-44 years | 12,110 (4.2%) | 502 (3.7%) | 1,430 (3.9%) | 3,237 (3.9%) | 4,159 (4.3%) | 2,782 (4.8%) |  |
| 45-64 years | 105,661 (37%) | 4,979 (36%) | 12,820 (35%) | 31,062 (38%) | 35,349 (37%) | 21,451 (37%) |  |
| 65-84 years | 137,674 (48%) | 6,867 (50%) | 18,370 (50%) | 39,484 (48%) | 45,853 (48%) | 27,100 (47%) |  |
| 85+ years | 31,803 (11%) | 1,370 (10.0%) | 3,923 (11%) | 9,010 (11%) | 11,161 (12%) | 6,339 (11%) |  |
| **Sex** |  |  |  |  |  |  | 0.013 |
| Male | 162,387 (57%) | 7,878 (57%) | 20,849 (57%) | 46,605 (56%) | 54,625 (57%) | 32,430 (56%) |  |
| Female | 124,861 (43%) | 5,840 (43%) | 15,694 (43%) | 36,188 (44%) | 41,897 (43%) | 25,242 (44%) |  |
| **Race** |  |  |  |  |  |  | <0.001 |
| White | 185,450 (65%) | 13,394 (98%) | 31,579 (86%) | 60,173 (73%) | 56,396 (58%) | 23,908 (41%) |  |
| Hispanic | 37,956 (13%) | 61 (0.4%) | 903 (2.5%) | 3,720 (4.5%) | 15,787 (16%) | 17,485 (30%) |  |
| Black | 34,239 (12%) | 192 (1.4%) | 3,329 (9.1%) | 15,972 (19%) | 9,018 (9.3%) | 5,728 (9.9%) |  |
| Asian or Pacific Islander | 26,267 (9.1%) | 23 (0.2%) | 408 (1.1%) | 2,184 (2.6%) | 13,895 (14%) | 9,757 (17%) |  |
| Native American | 1,866 (0.6%) | 16 (0.1%) | 190 (0.5%) | 422 (0.5%) | 858 (0.9%) | 380 (0.7%) |  |
| Unknown | 1,470 (0.5%) | 32 (0.2%) | 134 (0.4%) | 322 (0.4%) | 568 (0.6%) | 414 (0.7%) |  |
| **Region** |  |  |  |  |  |  | <0.001 |
| Midwest | 26,674 (9.3%) | 4,031 (29%) | 4,658 (13%) | 17,916 (22%) | 69 (<0.1%) | 0 (0%) |  |
| Northeast | 45,747 (16%) | 0 (0%) | 8,963 (25%) | 18,373 (22%) | 16,414 (17%) | 1,997 (3.5%) |  |
| South | 66,701 (23%) | 9,642 (70%) | 16,210 (44%) | 29,704 (36%) | 10,419 (11%) | 726 (1.3%) |  |
| West | 148,126 (52%) | 45 (0.3%) | 6,712 (18%) | 16,800 (20%) | 69,620 (72%) | 54,949 (95%) |  |
| **Primary Site** |  |  |  |  |  |  | <0.001 |
| Anus | 7,274 (2.5%) | 334 (2.4%) | 924 (2.5%) | 2,266 (2.7%) | 2,510 (2.6%) | 1,240 (2.2%) |  |
| Biliary Tract | 10,510 (3.7%) | 454 (3.3%) | 1,329 (3.6%) | 2,840 (3.4%) | 3,605 (3.7%) | 2,282 (4.0%) |  |
| Colon | 97,990 (34%) | 5,513 (40%) | 13,065 (36%) | 28,540 (34%) | 31,798 (33%) | 19,074 (33%) |  |
| Esophagus | 16,276 (5.7%) | 880 (6.4%) | 2,510 (6.9%) | 5,249 (6.3%) | 5,125 (5.3%) | 2,512 (4.4%) |  |
| Gallbladder | 4,550 (1.6%) | 185 (1.3%) | 488 (1.3%) | 1,122 (1.4%) | 1,632 (1.7%) | 1,123 (1.9%) |  |
| Gastroesophageal Junction | 7,961 (2.8%) | 378 (2.8%) | 1,151 (3.1%) | 2,407 (2.9%) | 2,556 (2.6%) | 1,469 (2.5%) |  |
| Gastrointestinal, Other | 3,097 (1.1%) | 99 (0.7%) | 374 (1.0%) | 923 (1.1%) | 1,047 (1.1%) | 654 (1.1%) |  |
| Liver | 31,105 (11%) | 984 (7.2%) | 3,126 (8.6%) | 8,277 (10.0%) | 11,440 (12%) | 7,278 (13%) |  |
| Pancreas, Other | 13,369 (4.7%) | 612 (4.5%) | 1,839 (5.0%) | 3,838 (4.6%) | 4,534 (4.7%) | 2,546 (4.4%) |  |
| Pancreatic Body & Tail | 13,860 (4.8%) | 609 (4.4%) | 1,804 (4.9%) | 4,291 (5.2%) | 4,629 (4.8%) | 2,527 (4.4%) |  |
| Pancreatic Head | 22,569 (7.9%) | 985 (7.2%) | 2,966 (8.1%) | 7,029 (8.5%) | 7,472 (7.7%) | 4,117 (7.1%) |  |
| Rectum | 40,351 (14%) | 2,147 (16%) | 5,275 (14%) | 11,334 (14%) | 13,668 (14%) | 7,927 (14%) |  |
| Small Intestine | 3,075 (1.1%) | 138 (1.0%) | 378 (1.0%) | 950 (1.1%) | 1,039 (1.1%) | 570 (1.0%) |  |
| Stomach | 15,261 (5.3%) | 400 (2.9%) | 1,314 (3.6%) | 3,727 (4.5%) | 5,467 (5.7%) | 4,353 (7.5%) |  |
| **TNM/AJCC Combined Stage** |  |  |  |  |  |  | <0.001 |
| Stage I-III | 175,818 (67%) | 8,655 (69%) | 22,349 (67%) | 50,996 (66%) | 59,084 (67%) | 34,734 (67%) |  |
| Stage IV & Above | 86,936 (33%) | 3,880 (31%) | 11,189 (33%) | 25,888 (34%) | 28,722 (33%) | 17,257 (33%) |  |
| **Primary Surgery Performed** |  |  |  |  |  |  | <0.001 |
| No Surgery | 130,562 (47%) | 5,429 (42%) | 16,067 (46%) | 37,815 (47%) | 44,347 (48%) | 26,904 (48%) |  |
| Surgery | 146,487 (53%) | 7,539 (58%) | 19,050 (54%) | 42,578 (53%) | 48,638 (52%) | 28,682 (52%) |  |
| **Radiation Therapy Performed** |  |  |  |  |  |  | <0.001 |
| No Therapy | 237,145 (83%) | 11,181 (82%) | 29,415 (80%) | 67,224 (81%) | 80,080 (83%) | 49,245 (85%) |  |
| Therapy | 50,103 (17%) | 2,537 (18%) | 7,128 (20%) | 15,569 (19%) | 16,442 (17%) | 8,427 (15%) |  |
| **Chemotherapy Performed** |  |  |  |  |  |  | <0.001 |
| No Therapy | 163,426 (57%) | 7,727 (56%) | 20,269 (55%) | 45,977 (56%) | 54,932 (57%) | 34,521 (60%) |  |
| Therapy | 123,822 (43%) | 5,991 (44%) | 16,274 (45%) | 36,816 (44%) | 41,590 (43%) | 23,151 (40%) |  |
| **Vital Status on Last Follow-up** |  |  |  |  |  |  | <0.001 |
| Alive | 153,472 (53%) | 7,291 (53%) | 19,183 (52%) | 43,854 (53%) | 52,376 (54%) | 30,768 (53%) |  |
| Dead | 133,776 (47%) | 6,427 (47%) | 17,360 (48%) | 38,939 (47%) | 44,146 (46%) | 26,904 (47%) |  |
