# Supplementary material for: Associations of Social Vulnerability and Race‐Ethnicity With Gastrointestinal Cancers in the United States
Source: Cancer Med. 2025 Mar 5;14(5):e70591. doi: 10.1002/cam4.70591 (PMC11880827; doi:10.1002/cam4.70591)
Supplement: Supplementary file 10 — Table S3. Patient Characteristics by Household Composition Status SVI Score. [file CAM4-14-e70591-s001.docx]

|  | **Household Composition SVI Subscore** | | | | | |  |
| --- | --- | --- | --- | --- | --- | --- | --- |
| **Characteristic** | **Overall**, N = 287248 (100%) | **0.000-0.199**, N = 4095 (1.4%) | **0.200-0.399**, N = 123403 (43%) | **0.400-0.599**, N = 95275 (33%) | **0.600-0.799**, N = 55775 (19%) | **0.800-0.999**, N = 8700 (3.0%) | **p-value** |
| **Age** |  |  |  |  |  |  | <0.001 |
| 20-44 years | 12,110 (4.2%) | 177 (4.3%) | 5,549 (4.5%) | 3,868 (4.1%) | 2,184 (3.9%) | 332 (3.8%) |  |
| 45-64 years | 105,661 (37%) | 1,515 (37%) | 44,249 (36%) | 35,283 (37%) | 21,252 (38%) | 3,362 (39%) |  |
| 65-84 years | 137,674 (48%) | 1,863 (45%) | 58,610 (47%) | 45,942 (48%) | 26,988 (48%) | 4,271 (49%) |  |
| 85+ years | 31,803 (11%) | 540 (13%) | 14,995 (12%) | 10,182 (11%) | 5,351 (9.6%) | 735 (8.4%) |  |
| **Sex** |  |  |  |  |  |  | <0.001 |
| Male | 162,387 (57%) | 2,384 (58%) | 68,872 (56%) | 54,066 (57%) | 32,033 (57%) | 5,032 (58%) |  |
| Female | 124,861 (43%) | 1,711 (42%) | 54,531 (44%) | 41,209 (43%) | 23,742 (43%) | 3,668 (42%) |  |
| **Race** |  |  |  |  |  |  | <0.001 |
| White | 185,450 (65%) | 2,011 (49%) | 71,711 (58%) | 66,082 (69%) | 39,005 (70%) | 6,641 (76%) |  |
| Hispanic | 37,956 (13%) | 358 (8.7%) | 20,767 (17%) | 11,185 (12%) | 5,168 (9.3%) | 478 (5.5%) |  |
| Black | 34,239 (12%) | 330 (8.1%) | 11,227 (9.1%) | 12,106 (13%) | 9,303 (17%) | 1,273 (15%) |  |
| Asian or Pacific Islander | 26,267 (9.1%) | 1,360 (33%) | 18,457 (15%) | 4,803 (5.0%) | 1,614 (2.9%) | 33 (0.4%) |  |
| Native American | 1,866 (0.6%) | 20 (0.5%) | 426 (0.3%) | 628 (0.7%) | 538 (1.0%) | 254 (2.9%) |  |
| Unknown | 1,470 (0.5%) | 16 (0.4%) | 815 (0.7%) | 471 (0.5%) | 147 (0.3%) | 21 (0.2%) |  |
| **Region** |  |  |  |  |  |  | <0.001 |
| Midwest | 26,674 (9.3%) | 320 (7.8%) | 5,026 (4.1%) | 8,699 (9.1%) | 12,476 (22%) | 153 (1.8%) |  |
| Northeast | 45,747 (16%) | 470 (11%) | 26,085 (21%) | 18,565 (19%) | 627 (1.1%) | 0 (0%) |  |
| South | 66,701 (23%) | 0 (0%) | 10,846 (8.8%) | 23,468 (25%) | 25,474 (46%) | 6,913 (79%) |  |
| West | 148,126 (52%) | 3,305 (81%) | 81,446 (66%) | 44,543 (47%) | 17,198 (31%) | 1,634 (19%) |  |
| **Primary Site** |  |  |  |  |  |  | <0.001 |
| Anus | 7,274 (2.5%) | 144 (3.5%) | 3,035 (2.5%) | 2,581 (2.7%) | 1,317 (2.4%) | 197 (2.3%) |  |
| Biliary Tract | 10,510 (3.7%) | 181 (4.4%) | 4,878 (4.0%) | 3,289 (3.5%) | 1,890 (3.4%) | 272 (3.1%) |  |
| Colon | 97,990 (34%) | 1,223 (30%) | 40,992 (33%) | 32,545 (34%) | 19,930 (36%) | 3,300 (38%) |  |
| Esophagus | 16,276 (5.7%) | 200 (4.9%) | 6,313 (5.1%) | 5,734 (6.0%) | 3,493 (6.3%) | 536 (6.2%) |  |
| Gallbladder | 4,550 (1.6%) | 62 (1.5%) | 2,196 (1.8%) | 1,370 (1.4%) | 801 (1.4%) | 121 (1.4%) |  |
| Gastroesophageal Junction | 7,961 (2.8%) | 104 (2.5%) | 3,415 (2.8%) | 2,706 (2.8%) | 1,518 (2.7%) | 218 (2.5%) |  |
| Gastrointestinal, Other | 3,097 (1.1%) | 32 (0.8%) | 1,441 (1.2%) | 1,013 (1.1%) | 538 (1.0%) | 73 (0.8%) |  |
| Liver | 31,105 (11%) | 655 (16%) | 13,339 (11%) | 10,453 (11%) | 5,821 (10%) | 837 (9.6%) |  |
| Pancreas, Other | 13,369 (4.7%) | 157 (3.8%) | 5,802 (4.7%) | 4,517 (4.7%) | 2,505 (4.5%) | 388 (4.5%) |  |
| Pancreatic Body & Tail | 13,860 (4.8%) | 203 (5.0%) | 6,187 (5.0%) | 4,540 (4.8%) | 2,568 (4.6%) | 362 (4.2%) |  |
| Pancreatic Head | 22,569 (7.9%) | 301 (7.4%) | 9,706 (7.9%) | 7,590 (8.0%) | 4,359 (7.8%) | 613 (7.0%) |  |
| Rectum | 40,351 (14%) | 540 (13%) | 16,960 (14%) | 13,527 (14%) | 8,008 (14%) | 1,316 (15%) |  |
| Small Intestine | 3,075 (1.1%) | 30 (0.7%) | 1,370 (1.1%) | 1,040 (1.1%) | 550 (1.0%) | 85 (1.0%) |  |
| Stomach | 15,261 (5.3%) | 263 (6.4%) | 7,769 (6.3%) | 4,370 (4.6%) | 2,477 (4.4%) | 382 (4.4%) |  |
| **TNM/AJCC Combined Stage** |  |  |  |  |  |  | 0.053 |
| Stage I-III | 175,818 (67%) | 2,580 (67%) | 75,118 (67%) | 58,712 (67%) | 34,080 (67%) | 5,328 (68%) |  |
| Stage IV & Above | 86,936 (33%) | 1,249 (33%) | 37,268 (33%) | 28,729 (33%) | 17,160 (33%) | 2,530 (32%) |  |
| **Primary Surgery Performed** |  |  |  |  |  |  | <0.001 |
| No Surgery | 130,562 (47%) | 2,021 (51%) | 56,230 (47%) | 43,481 (47%) | 25,106 (47%) | 3,724 (45%) |  |
| Surgery | 146,487 (53%) | 1,954 (49%) | 63,083 (53%) | 48,469 (53%) | 28,459 (53%) | 4,522 (55%) |  |
| **Radiation Therapy Performed** |  |  |  |  |  |  | <0.001 |
| No Therapy | 237,145 (83%) | 3,419 (83%) | 103,068 (84%) | 78,017 (82%) | 45,520 (82%) | 7,121 (82%) |  |
| Therapy | 50,103 (17%) | 676 (17%) | 20,335 (16%) | 17,258 (18%) | 10,255 (18%) | 1,579 (18%) |  |
| **Chemotherapy Performed** |  |  |  |  |  |  | <0.001 |
| No Therapy | 163,426 (57%) | 2,325 (57%) | 70,481 (57%) | 53,645 (56%) | 31,875 (57%) | 5,100 (59%) |  |
| Therapy | 123,822 (43%) | 1,770 (43%) | 52,922 (43%) | 41,630 (44%) | 23,900 (43%) | 3,600 (41%) |  |
| **Vital Status on Last Follow-up** |  |  |  |  |  |  | <0.001 |
| Alive | 153,472 (53%) | 2,220 (54%) | 67,717 (55%) | 50,760 (53%) | 28,393 (51%) | 4,382 (50%) |  |
| Dead | 133,776 (47%) | 1,875 (46%) | 55,686 (45%) | 44,515 (47%) | 27,382 (49%) | 4,318 (50%) |  |
