# Supplementary material for: Associations of Social Vulnerability and Race‐Ethnicity With Gastrointestinal Cancers in the United States
Source: Cancer Med. 2025 Mar 5;14(5):e70591. doi: 10.1002/cam4.70591 (PMC11880827; doi:10.1002/cam4.70591)
Supplement: Supplementary file 11 — Table S4. Patient Characteristics by Housing‐Transportation Status SVI Score. [file CAM4-14-e70591-s004.docx]

|  | **Housing-Transport SVI Subscore** | | | | | |  |
| --- | --- | --- | --- | --- | --- | --- | --- |
| **Characteristic** | **Overall**, N = 287248 (100%) | **0.000-0.199**, N = 571 (0.2%) | **0.200-0.399**, N = 45081 (16%) | **0.400-0.599**, N = 158349 (55%) | **0.600-0.799**, N = 81449 (28%) | **0.800-0.999**, N = 1798 (0.6%) | **p-value** |
| **Age** |  |  |  |  |  |  | <0.001 |
| 20-44 years | 12,110 (4.2%) | 30 (5.3%) | 1,852 (4.1%) | 6,612 (4.2%) | 3,541 (4.3%) | 75 (4.2%) |  |
| 45-64 years | 105,661 (37%) | 203 (36%) | 16,169 (36%) | 58,462 (37%) | 30,097 (37%) | 730 (41%) |  |
| 65-84 years | 137,674 (48%) | 296 (52%) | 21,988 (49%) | 75,691 (48%) | 38,830 (48%) | 869 (48%) |  |
| 85+ years | 31,803 (11%) | 42 (7.4%) | 5,072 (11%) | 17,584 (11%) | 8,981 (11%) | 124 (6.9%) |  |
| **Sex** |  |  |  |  |  |  | 0.007 |
| Male | 162,387 (57%) | 338 (59%) | 25,282 (56%) | 89,484 (57%) | 46,210 (57%) | 1,073 (60%) |  |
| Female | 124,861 (43%) | 233 (41%) | 19,799 (44%) | 68,865 (43%) | 35,239 (43%) | 725 (40%) |  |
| **Race** |  |  |  |  |  |  | <0.001 |
| White | 185,450 (65%) | 509 (89%) | 36,492 (81%) | 101,972 (64%) | 45,418 (56%) | 1,059 (59%) |  |
| Hispanic | 37,956 (13%) | 23 (4.0%) | 2,341 (5.2%) | 19,272 (12%) | 16,166 (20%) | 154 (8.6%) |  |
| Black | 34,239 (12%) | 12 (2.1%) | 4,515 (10%) | 20,914 (13%) | 8,446 (10%) | 352 (20%) |  |
| Asian or Pacific Islander | 26,267 (9.1%) | 24 (4.2%) | 1,401 (3.1%) | 14,463 (9.1%) | 10,372 (13%) | 7 (0.4%) |  |
| Native American | 1,866 (0.6%) | 2 (0.4%) | 158 (0.4%) | 882 (0.6%) | 602 (0.7%) | 222 (12%) |  |
| Unknown | 1,470 (0.5%) | 1 (0.2%) | 174 (0.4%) | 846 (0.5%) | 445 (0.5%) | 4 (0.2%) |  |
| **Region** |  |  |  |  |  |  | <0.001 |
| Midwest | 26,674 (9.3%) | 0 (0%) | 8,986 (20%) | 15,783 (10.0%) | 1,827 (2.2%) | 78 (4.3%) |  |
| Northeast | 45,747 (16%) | 0 (0%) | 17,148 (38%) | 26,602 (17%) | 1,997 (2.5%) | 0 (0%) |  |
| South | 66,701 (23%) | 555 (97%) | 14,168 (31%) | 36,297 (23%) | 14,466 (18%) | 1,215 (68%) |  |
| West | 148,126 (52%) | 16 (2.8%) | 4,779 (11%) | 79,667 (50%) | 63,159 (78%) | 505 (28%) |  |
| **Primary Site** |  |  |  |  |  |  | <0.001 |
| Anus | 7,274 (2.5%) | 27 (4.7%) | 1,122 (2.5%) | 4,085 (2.6%) | 2,020 (2.5%) | 20 (1.1%) |  |
| Biliary Tract | 10,510 (3.7%) | 22 (3.9%) | 1,648 (3.7%) | 5,679 (3.6%) | 3,113 (3.8%) | 48 (2.7%) |  |
| Colon | 97,990 (34%) | 198 (35%) | 15,484 (34%) | 54,111 (34%) | 27,478 (34%) | 719 (40%) |  |
| Esophagus | 16,276 (5.7%) | 39 (6.8%) | 2,801 (6.2%) | 9,203 (5.8%) | 4,140 (5.1%) | 93 (5.2%) |  |
| Gallbladder | 4,550 (1.6%) | 5 (0.9%) | 650 (1.4%) | 2,479 (1.6%) | 1,386 (1.7%) | 30 (1.7%) |  |
| Gastroesophageal Junction | 7,961 (2.8%) | 11 (1.9%) | 1,411 (3.1%) | 4,348 (2.7%) | 2,158 (2.6%) | 33 (1.8%) |  |
| Gastrointestinal, Other | 3,097 (1.1%) | 4 (0.7%) | 491 (1.1%) | 1,626 (1.0%) | 965 (1.2%) | 11 (0.6%) |  |
| Liver | 31,105 (11%) | 41 (7.2%) | 3,892 (8.6%) | 17,503 (11%) | 9,496 (12%) | 173 (9.6%) |  |
| Pancreas, Other | 13,369 (4.7%) | 16 (2.8%) | 2,298 (5.1%) | 7,357 (4.6%) | 3,616 (4.4%) | 82 (4.6%) |  |
| Pancreatic Body & Tail | 13,860 (4.8%) | 30 (5.3%) | 2,426 (5.4%) | 7,650 (4.8%) | 3,674 (4.5%) | 80 (4.4%) |  |
| Pancreatic Head | 22,569 (7.9%) | 58 (10%) | 3,864 (8.6%) | 12,436 (7.9%) | 6,096 (7.5%) | 115 (6.4%) |  |
| Rectum | 40,351 (14%) | 88 (15%) | 6,450 (14%) | 22,156 (14%) | 11,389 (14%) | 268 (15%) |  |
| Small Intestine | 3,075 (1.1%) | 9 (1.6%) | 594 (1.3%) | 1,668 (1.1%) | 787 (1.0%) | 17 (0.9%) |  |
| Stomach | 15,261 (5.3%) | 23 (4.0%) | 1,950 (4.3%) | 8,048 (5.1%) | 5,131 (6.3%) | 109 (6.1%) |  |
| **TNM/AJCC Combined Stage** |  |  |  |  |  |  | <0.001 |
| Stage I-III | 175,818 (67%) | 370 (68%) | 27,582 (66%) | 97,669 (67%) | 49,155 (67%) | 1,042 (66%) |  |
| Stage IV & Above | 86,936 (33%) | 175 (32%) | 14,057 (34%) | 47,594 (33%) | 24,563 (33%) | 547 (34%) |  |
| **Primary Surgery Performed** |  |  |  |  |  |  | <0.001 |
| No Surgery | 130,562 (47%) | 242 (43%) | 20,008 (46%) | 72,091 (47%) | 37,450 (48%) | 771 (45%) |  |
| Surgery | 146,487 (53%) | 317 (57%) | 23,577 (54%) | 80,728 (53%) | 40,935 (52%) | 930 (55%) |  |
| **Radiation Therapy Performed** |  |  |  |  |  |  | <0.001 |
| No Therapy | 237,145 (83%) | 454 (80%) | 36,360 (81%) | 130,347 (82%) | 68,447 (84%) | 1,537 (85%) |  |
| Therapy | 50,103 (17%) | 117 (20%) | 8,721 (19%) | 28,002 (18%) | 13,002 (16%) | 261 (15%) |  |
| **Chemotherapy Performed** |  |  |  |  |  |  | <0.001 |
| No Therapy | 163,426 (57%) | 275 (48%) | 24,286 (54%) | 89,567 (57%) | 48,231 (59%) | 1,067 (59%) |  |
| Therapy | 123,822 (43%) | 296 (52%) | 20,795 (46%) | 68,782 (43%) | 33,218 (41%) | 731 (41%) |  |
| **Vital Status on Last Follow-up** |  |  |  |  |  |  | <0.001 |
| Alive | 153,472 (53%) | 318 (56%) | 24,682 (55%) | 84,607 (53%) | 42,973 (53%) | 892 (50%) |  |
| Dead | 133,776 (47%) | 253 (44%) | 20,399 (45%) | 73,742 (47%) | 38,476 (47%) | 906 (50%) |  |
