# Supplementary material for: Associations of Social Vulnerability and Race‐Ethnicity With Gastrointestinal Cancers in the United States
Source: Cancer Med. 2025 Mar 5;14(5):e70591. doi: 10.1002/cam4.70591 (PMC11880827; doi:10.1002/cam4.70591)
Supplement: Supplementary file 12 — Data S1. [file CAM4-14-e70591-s011.docx]

**METHODS**

**Data Sources**

Per the methodologies described by the CDC-SVI development team, the 15 social factors are grouped into the 4 SDoH themes of SES (poverty, unemployment, income level, and high school diploma status), ML (minoritized racial and ethnic group [American Indian and Alaska Native, Asian, Black or African American, American Indian and Alaska Native, Asian, Native Hawaiian or other Pacific Islander, other, and multiracial (per the US Census) and proficiency with English), HH (household members aged ≥65 and ≤17 years, disability status, single-parent status), and HT (multiunit structure, mobile homes, crowding, no vehicle, group quarters) were assigned weighted-average scores based on the relative proportional makeup of the census populations. These themed scores were ascribed from a range of 0 to 1, representing the relative comparisons across the whole range of standardized census tract areas (e.g., a score of 0.7 represents a census tract being more socially vulnerable in a specific SDoH category than 70% of census tracts in the country). A total composite from each of these four themes encompasses a dynamic, differentially weighted average based on internal modeling schemes of sociodemographic contextualization (e.g. Census Tract A could have its Total SVI score calculated based on 35% of the SES-theme, 15% of the ML-theme, 23% of HH-theme, 27% of HT-theme; Census Tract B could be comprised of 33%, 17%, 22%, 28%, respectively; Census Tract C could be 25%, 25%, 25%, 25%, respectively; and etc.) that are not made publicly available. Based on these formulaic manipulations, the total SVI scores also range from 0 to 1 and represent a similar interpretation of the SVI-theme scores (e.g., a score of 0.6 represents a census tract being more socially vulnerable across the total composite of SDoH-themes than 60% of census tracts in the country). In order to upcode the categorizations of census tracts to counties, for which census tracts are designated to not overlap into more than one county classification (e.g., Census tracts A, B, C only belong to County A and **cannot** be a part of Counties B, C, etc.), scores that are assigned to the level of counties comprise the population-weighted averages of all the census tracts comprising a specific county. These scores also remain ranged from 0 to 1 and hold similar interpretations as prior census tracts for the total SVI composite and SVI-themes. Upcoding to county-level scores were necessitated due to the level of geocode/geography available within the selected SEER dataset in order to remain compliant with HIPAA and data use agreement standards set by the SEER administrators.

Based on the total SVI’s representation as a dynamic, differentially weighted composite encompassing these themes, univariate regressions were elected to allow interpretations of the total SVI to preserve its real-world contextualized, dynamic differential-weights and, given its summated and composited formulation, retain a multivariate-interpretation rather than a multivariate analysis in concert with the SVI-theme factors (i.e. univariate model of dependent variate outcome and independent variate Total vs. multivariate model of dependent variate outcome, independent covariates of Total SVI, SES, ML, HH, and HT). In other words, the multivariate approach would reassign weights of 20% per category and remove the real-world valences already built into the total SVI measure. Univariate modeling for each of the SVI-themes is also justified by allowing independent delineation of how much certain SDoH-themes influence the associations of the total SVI composite by isolating its true value association without confounding by the dynamic weights associated with total SVI calculation (e.g., if Census Tract A has a weight of 20% for ML but Census Tract B has a weight of 30% for ML, we would not know how much ML in isolation confers its influence on either Census Tract’s Total SVI/overall SDoH-composite effects in multivariate analyses because of the proprietary/inaccessible nature of the dynamic weights of the total SVI by the CDC).

**Variables**

Months under surveillance was a length-of-care measurement reflecting the period of active follow-up that a patient received related to their primary malignancy. Staging was based on SEER-designated variables and recoded based on American Joint Committee on Cancer, 6^th^ Edition (AJCC-6) classifications. Stage at presentation was categorized as early-stage (I, II, III) versus advanced stage (IV). Outcomes of interest included chemotherapy, radiation therapy, and utilization of surgical resection of the GIC SEER-provided variables were used to define receipt of chemotherapy, radiation therapy, and/or surgical resection.

*Statistical Methods:*

In re-normalizing the extent of SVI effects to the pertinent GIC patient population, total and subtheme SVI scores of each patient cohort were split into relative equivalently sampled quintiles on a per-histology/subtype level. This allows relevancy of SDoH-range to the patient population at hand in the specific comparisons outlined in this investigation’s design (in other words, how does SDoH-vulnerability vary within GIC populations and what is their influence relative to the span of patient clinicodemographics represented). Quintiles were ranked as discrete variate levels (with reference level set to the lowest vulnerability quintile) within univariate logistic regression models and utilized to assess for associated occurrences of whether patients received a certain treatment modality, as well as whether they had advanced staging on preliminary presentation based on SEER-staging/AJCC-TNM-staging classifications available in the selected SEER dataset.

**RESULTS**

14 GICs demonstrated decreased odds of receiving chemotherapy (lowest, pancreas-OR, 0.90; 95%CI, 0.88-0.93), radiotherapy (hepatic-OR, 0.87; 95%CI, 0.85-0.89) and surgical resection (esophagus-OR, 0.90; 95%CI, 0.87-0.92) for 13/14, 10/14, and 8/14 GIC types, respectively

**Impact of SVI on Treatment of GIC**

Increasing total SVI was associated with decreased odds of surgical resection for 8/14 GIC types (esophagus: OR, 0.90; 95% CI, 0.87-0.92; liver, 0.91; 95% CI, 0.89-0.93; pancreas head: OR, 0.96; 95% CI, 0.94-0.98; biliary tract: OR, 0.97; 95% CI, 0.93-0.99; rectum: OR, 0.95; 95% CI, 0.94-0.97; all p<0.001).

Increasing total SVI was associated with decreased odds of chemotherapy for 13/14 GIC types (esophagus: OR, 0.93; 95% CI, 0.90-0.95; gastroesophageal junction: OR, 0.94; 95% CI, 0.91-0.98; stomach: OR, 0.94; 95% CI, 0.91-0.96; pancreas head: OR, 0.91; 95% CI, 0.90-0.93; biliary tract: OR, 0.91; 95% CI, 0.89-0.94; gallbladder: OR, 0.94; 95% CI, 0.87-0.95; small intestine: OR, 0.91; 95%CI, 0.87-0.96; colon: OR, 0.97; 95%CI, 0.96-0.98; all p<0.001).

Increasing total SVI was associated with decreased odds of radiation therapy for 10/14 GIC types (esophagus: OR, 0.96; 95% CI, 0.93-0.98; gastroesophageal junction: 0.93; 95% CI, 0.90-0.96; stomach: OR, 0.94; 95% CI, 0.91-0.97; liver: 0.87; 95% CI, 0.85-0.89; pancreas head: 0.95; 95% CI, 0.92-0.97; biliary tract: 0.93; 95% CI, 0.89-0.97; anus: OR, 0.91; 95% CI, 0.88-0.94; all p<0.001)

When comparing Non-White and White patients with increasing total SVI, Non-White patients were more likely to not receive surgery than White patients for 8/14 GIC types: colon (Non-White, OR 0.97, 95%CI 0.95-0.99; Non-White, OR 1.01, 95%CI 1.00-1.03), esophagus (Non-White, OR 0.90, 95%CI 0.85-0.96; White, OR 0.93, 95%CI 0.91-0.96), gastroesophageal junction (Non-White, OR 0.92, 95%CI 0.86-0.99; White, OR 1.00, 95%CI 0.97-1.04), hepatic (Non-White, OR 0.88, 95%CI 0.86-0.91; White, OR 0.91, 95%CI 0.89-0.94), pancreas – head (Non-White, OR 0.94, 95%CI 0.90-0.98; White, OR 0.97, 95%CI 0.95-0.99), pancreas – body & tail (Non-White, OR 0.93, 95%CI 0.89-0.98; White, OR 0.97, 95%CI 0.94-1.01), rectum (Non-White, OR 0.95, 95%CI 0.92-0.97; White, OR 0.97, 95%CI 0.95-0.99), and gastric primary sites (Non-White, OR 0.95, 95%CI 0.92-0.98; White, OR 1.00, 95%CI 0.96-1.04).

When comparing Non-White and White patients with increasing total SVI, Non-White patients were more likely to not receive radiation therapy for 7/14 GIC types: anus (Non-White, OR 0.88, 95%CI 0.82-0.95; White, OR 0.93, 95%CI 0.90-0.99), biliary tract (Non-White, OR 0.88, 95%CI 0.82-0.94; White, OR 0.97, 95%CI 0.92-1.02), pancreas – head (Non-White, OR 0.92, 95%CI 0.87-0.96; White, OR 0.97, 95%CI 0.94-1.00), pancreas – body & tail (Non-White, OR 0.92, 95%CI 0.85-0.99; White, OR 0.95, 95%CI 0.90-0.99), pancreas – other (Non-White, OR 0.90, 95%CI 0.82-0.98; White, OR 0.94, 95%CI 0.88-0.99), gastric (Non-White, OR 0.91, 95%CI 0.87-0.95; White, OR 0.96, 95%CI 0.91-1.01), gastrointestinal-other (Non-White, OR 0.76, 95%CI 0.63-0.90; White, 0.94, 95%CI 0.83-1.06).

In contrast to surgery and radiation therapy, both non-White and White patients displayed similar magnitudes of decreased chemotherapy receipt with increasing total SVI for 6/14 GIC sites: esophagus (Non-White, OR 0.92, 95%CI 0.88-0.96; White, OR 0.94, 95%CI 0.91-0.96), gallbladder (Non-White, OR 0.90, 95%CI 0.85-0.96; White, OR 0.90, 95%CI 0.85-0.96), pancreas – head (Non-White, OR 0.92, 95%CI 0.89-0.95; White, OR 0.93, 95%CI 0.90-0.95), pancreas – body & tail (Non-White, OR 0.91, 95%CI 0.87-0.94; White, OR 0.92, 95%CI 0.90-0.95), small intestine (Non-White, OR 0.91, 95%CI 0.84-0.99; White, OR 0.93, 95%CI 0.87-0.99), gastric (Non-White, OR 0.92, 95%CI 0.90-0.95; White, OR 0.92, 95%CI 0.89-0.96).
